# The type of carbon source not the growth rate it supports can determine diauxie in *Saccharomyces cerevisiae*

**DOI:** 10.1038/s42003-025-07747-z

**Published:** 2025-02-27

**Authors:** Yu Huo, Weronika Danecka, Iseabail Farquhar, Kim Mailliet, Tessa Moses, Edward W. J. Wallace, Peter S. Swain

**Affiliations:** 1 Centre for Engineering Biology, The University of Edinburgh, Edinburgh, United Kingdom; 2 School of Biological Sciences, The University of Edinburgh, Edinburgh, United Kingdom; 3 EdinOmics, RRID:SCR_021838, Centre for Engineering Biology, School of Biological Sciences, CH Waddington Building, The University of Edinburgh, Edinburgh, United Kingdom

**Keywords:** Fungal genetics, Biochemical networks

## Abstract

How cells choose between carbon sources is a classic example of cellular decision-making. Microbes often prioritise glucose, but there has been little investigation of whether other sugars are also preferred. Here we study budding yeast growing on mixtures of sugars with palatinose, a sucrose isomer that cells catabolise with the *MAL* regulon. We find that the decision-making involves more than carbon flux-sensing: yeast prioritise galactose over palatinose, but sucrose and fructose weakly if at all despite each allowing faster growth than palatinose. With genetic perturbations and transcriptomics, we show that the regulation is active with repression of the *MAL* genes via Gal4, the *GAL* regulon’s master regulator. We argue, using mathematical modelling, that cells enforce their preference for galactose through weakening the *MAL* regulon’s positive feedback. They do so through decreasing intracellular palatinose by repressing *MAL11*, the palatinose transporter, and expressing the isomaltases *IMA1* and *IMA5*. Supporting these predictions, we show that deleting *IMA1* abolishes diauxie. Our results demonstrate that budding yeast actively prioritises carbon sources other than glucose and that such priorities need not reflect differences in growth rates. They imply that carbon-sensing strategies even in model organisms are more complex than previously thought.

## Abstract (web summary)

A study on how budding yeast decides between carbon sources when growing on pairs of sugars suggests that cells can actively prioritise sugars other than glucose and that such priorities need not reflect differences in growth rates.

## Introduction

All cells respond to change. Understanding the strategies they use to do so is fundamental: we expect these strategies to be more deeply conserved than their biochemical implementations^1–3^, with different cell types realising the same strategy in different ways.

A classic example of decision-making is whether a cell consumes two available carbon sources either sequentially—often called diauxie^4^—or simultaneously. Both the bacterium *Escherichia coli* and the eukaryotic budding yeast *Saccharomyces cerevisiae*  prefer glucose over other carbon sources^5^, and at sufficient concentrations, cells specialise their physiology to its consumption. For budding yeast, cells both repress expression of genes for metabolising other carbon sources^6^ and remove any transporters for these carbon sources from the plasma membrane^7–10^. Yet apart from glucose, budding yeast can consume at least six other sugars^11^, and we know little about how or even whether cells discriminate between them.

We therefore do not have a clear picture of how budding yeast, one of the most studied eukaryotic cells, organise their carbon-sensing, a task that involves kinases conserved in metazoans^12^. Although much regulation is known to impose the cells’ preference for glucose, it is unclear if similar complexity exists to enforce a hierarchy of preferences for all pairs of sugars. Control might be more generic, perhaps through sensing of glycolytic flux as happens in *E. coli*^13,14^ or occurring passively through dilution because different sugars allow different growth rates^15^.

**Fig. 1:**
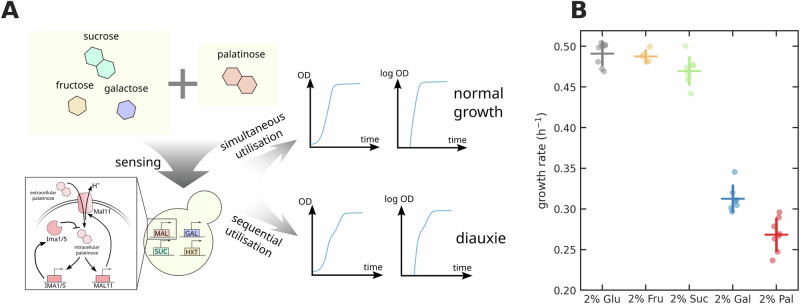
Cells either co- or sequentially consume two carbon sources and grow at different rates in single carbon sources. **A** We investigated whether cells of budding yeast exhibited diauxic growth, a hallmark of sequential consumption, in mixtures of palatinose with either fructose, sucrose, galactose, or, as a control, glucose. **B** Budding yeast has different mid-log growth rates on different sugars; palatinose supports the slowest growth. Specific growth rates in glucose, fructose or sucrose are significantly higher than those in galactose and palatinose (*p* < 10^−9^ using an independent samples t-test) and the rate in galactose higher than that in palatinose (*p* = 4.4 × 10^−4^). There are no significant differences between the growth rates of glucose, fructose, and sucrose (*p* > 1.7 × 10^−2^). Data are shown as mean  ± standard deviation of at least five biological replicates (dots).

Here we systematically investigate budding yeast’s decision-making on two sugars focusing on pairs that do not include glucose (Fig. 1A). Cells import these sugars in two ways, via either hexose transporters or proton symporters^11^. If the same transporters import both sugars, the sugars may compete to bind the transporters^16^. We therefore chose pairs of sugars that require both types of import mechanisms, reasoning that such sugars are more likely to be independently regulated.

For the sugar requiring proton symport, we focused on palatinose, a disaccharide of glucose and fructose (with an *α*-1,6 linkage), and a constituent of sugar cane and honey^17^. Palatinose is a substrate of the *MAL* regulon^17^ (Fig. 1A). The laboratory strain BY4741, and its prototrophic antecedent FY4, both grow on palatinose but not on the more studied maltose^17^, another disaccharide (two glucose molecules with an *α*-1,4 linkage) also imported by proton symporters. Palatinose is the only known substrate of these strains’ *MAL* regulons. The *MAL* regulon responsible has two transcriptional activators^18^, Mal13 and Znf1. These activators sense intracellular palatinose and induce expression of the *MAL11* palatinose transporter and two catabolic enzymes *IMA1* and *IMA5*^17^.

For the sugars requiring hexose transporters, we investigated fructose, galactose, sucrose (a disaccharide of glucose and fructose with an *α*-1,2 linkage) and, as a control, glucose^11^. Although these sugars support different growth rates (Fig. 1B), all feed upper glycolysis^19^. Cells convert glucose into glucose-6-phosphate, the entry point of glycolysis; fructose into fructose-6-phosphate, which is immediately downstream of glucose-6-phosphate; galactose into glucose-6-phosphate; and cleave palatinose and sucrose into their fructose and glucose constituents, palatinose intracellularly and sucrose predominately extracellularly.

We found that budding yeast has a sugar hierarchy beyond glucose. We observed diauxie in mixtures of galactose and palatinose, as well as for glucose and palatinose, but not in mixtures of fructose or sucrose with palatinose. Combining genetic perturbations and transcriptomics, we show that cells implement their preference for galactose both by repressing the expression of *MAL11*, encoding the palatinose transporter, and by expressing the isomaltases (*IMA1* and *IMA5*), the enzymes that catabolise palatinose. Our results point not towards generic carbon-sensing, but towards specific regulation that actively enforces a sugar hierarchy.

## Results

### Cells growing in galactose-palatinose mixtures show diauxie

We used plate readers to characterise the cells’ growth, measuring the optical density (OD) and for fluorescently tagged strains the fluorescence of cultures. With the omniplate software package^20^, we corrected for the nonlinear dependence of the OD on cell number^21^ and for autofluorescence^22^, used Gaussian processes to estimate growth rates over time^23^, and automatically extracted regions of exponential growth^24^.

**Fig. 2:**
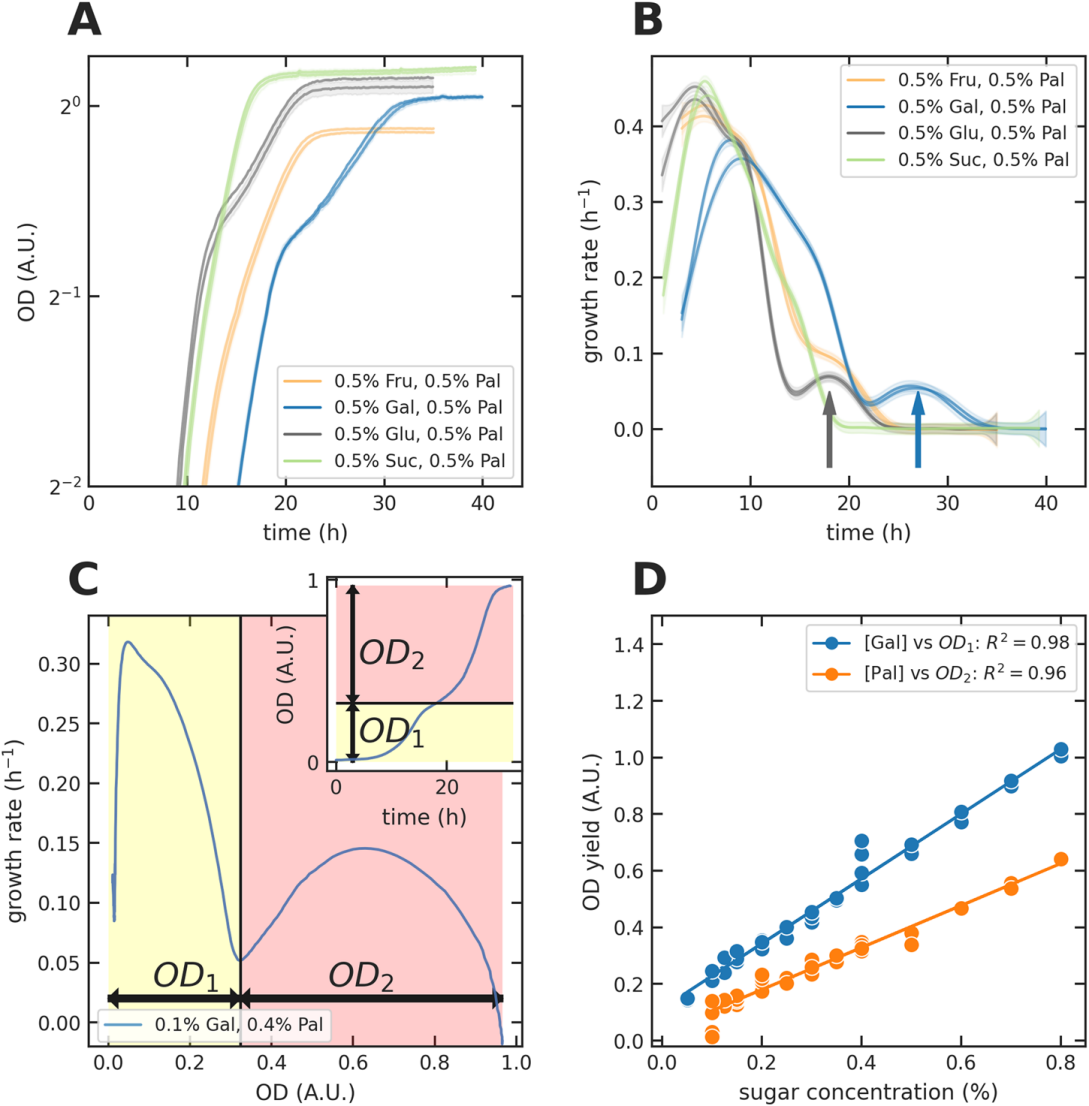
Cells consume galactose before palatinose. **A, B** We observed diauxie in the growth dynamics of the wild-type prototrophic strain (FY4) in galactose-palatinose mixtures, similar to that in glucose-palatinose mixtures. The arrows in **B** point to a second peak in the specific growth rate for these mixtures. We show two biological replicates for each set of concentrations; the shading gives the standard deviation of two technical replicates. **C** To quantify the OD yield of each growth phase, we found the local minimum of the specific growth rate between the two maxima. If this minimum marks the end of growth phase one and the beginning of growth phase two, then the OD yield of growth phase one (*OD*_1_) is the OD at the local minimum, which we denote *OD*_switch_, minus the initial OD, and the OD yield of growth phase two (*OD*_2_) is the difference between the final OD and *OD*_switch_. **D** In galactose-palatinose mixtures, the OD yield of growth phase one linearly correlated with galactose concentrations; the OD yield of growth phase two linearly correlated with palatinose concentrations. We found each data point using the method in **C**.

We observed clear diauxic-like growth for galactose-palatinose mixtures, similar to the expected diauxie^6,25^ that we also saw in glucose-palatinose mixtures (Fig. 2A). Consistent with cells sequentially using the two sugars, the growth rate had two local maxima (Fig. 2B), likely because cells only expressed the *MAL* regulon once galactose was exhausted generating a lag^26^. The minimum in the growth rate between the two maxima divides the growth curve into two phases. For the first phase, the galactose concentration determined the amount of growth; for the second phase, the palatinose concentration determined growth. We found the OD of the culture, OD_switch_, at the local minimum of the growth rate over time (Fig. 2C). We then defined the yields for the two growth periods: the difference between OD_switch_ and the initial OD for the first; and the difference between the final OD and OD_switch_ for the second. The first growth yield linearly correlated with the galactose concentration and the second with the palatinose concentration (Fig. 2D), as they did too for glucose-palatinose diauxie (Fig. S1).

**Fig. 3:**
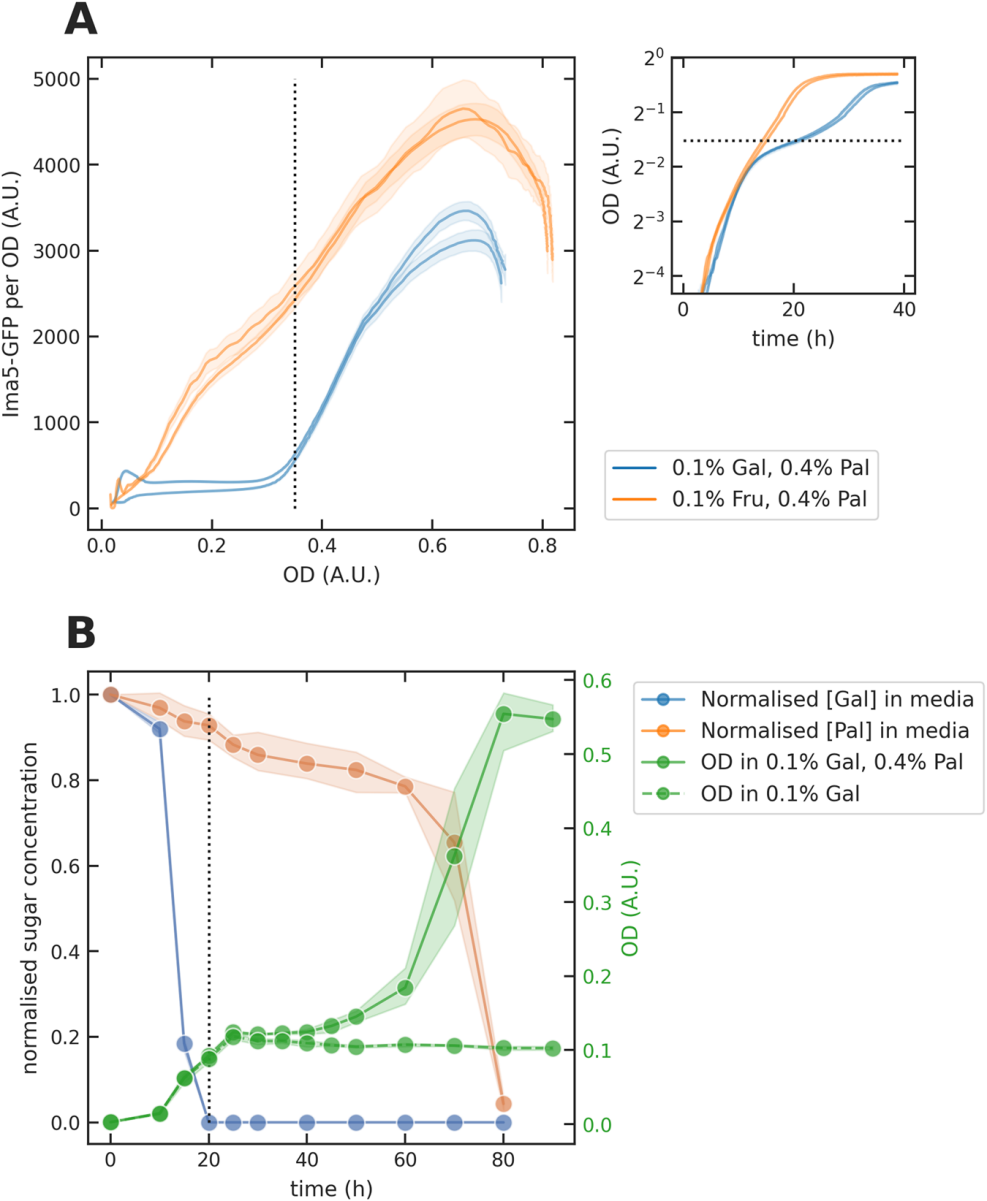
Extracellular palatinose decreases only after a delay in galactose-palatinose mixtures, and cells delay too in expressing isomaltases. **A** Cells expressed the isomaltase gene *IMA5* after a delay in galactose-palatinose, but immediately in fructose-palatinose mixtures. We show the level of isomaltase Ima5-GFP per OD as a function of OD in fructose- and galactose-palatinose mixtures for two biological replicates. Inset: the growth dynamics. The black dotted line marks the OD at which galactose is almost exhausted. **B** Metabolomics data confirmed that cells prioritise galactose over palatinose; the extracellular palatinose concentration only rapidly fell once extracellular galactose was exhausted. We measured the OD of the samples in a plate reader and the concentrations of extracellular galactose and palatinose by GC-MS, normalising by the values of the first time point (0 h). Each data point represents the mean of three biological replicates and the shaded area their standard deviation.

A characteristic feature of diauxic growth on glucose is that cells repress genes for catabolising other carbon sources^6^, and so we determined if the initial growth on galactose caused cells to repress the genes to catabolise palatinose. Cells use two isomaltase enzymes, Ima1 and Ima5, to cleave palatinose^17^. Focusing on *IMA5-GFP*, we observed that cells do repress *IMA5*, with levels of Ima5-GFP increasing only after the first phase of growth in galactose-palatinose mixtures (Fig. 3A). We confirmed this behaviour in single cells for *IMA1-GFP* (Fig. S2) and also that the galactose-palatinose diauxie depended neither on the sugar concentrations (Fig. S3) nor on the carbon source we used to pre-grow the cells (Fig. S4A--C). It was also not an artefact of cells consuming any ethanol or acetate generated by their growth on galactose (Fig. S4D).

Finally we grew cells in flasks and measured the extracellular galactose and palatinose concentrations over time using metabolomics^27^ (Fig. 3B). The galactose vanished within 20 hours when approximately 90% of the palatinose was still present, and the palatinose concentration only quickly decreased during the second phase of growth.

Galactose enables faster growth than palatinose, but fructose and sucrose enable growth that is even faster and similar to that on glucose (Fig. 1B). Yet we observed no obvious diauxie in fructose-palatinose or sucrose-palatinose mixtures (Fig. 2A). There was only a single maximum in the growth rate and cells immediately expressed the isomaltases (Figs. 3A, S2C, & S5). Nevertheless, we suspect that the behaviour is more subtle than simultaneous consumption—a point we will return to in the Discussion—because for some concentrations we observed a ‘shoulder’ in the growth rate versus time (Figs. 2B fructose-palatinose & S5).

Our results suggest a specific mechanism generating the galactose-palatinose diauxie. The different behaviour in fructose- and sucrose-palatinose mixtures is inconsistent with a general carbon flux-sensing mechanism because these two sugars likely generate a higher glycolytic flux than galactose: they support faster growth and all three sugars feed glycolysis. The higher growth rates of fructose and sucrose also rule out passive control through dilution^15^, where the rate of division enabled by one sugar causes low intracellular levels of enzymes for another, because enzymes are so quickly passed onto daughter cells.

### Active Gal4 limits the use of palatinose

To investigate how intracellular galactose represses *MAL* genes, we constitutively activated the *GAL* regulon. In the presence of galactose, the master transcriptional regulator Gal4 induces expression of *GAL* genes; in the absence of galactose, Gal4 is inactivated by another transcription factor Gal80^28^. Deleting the *GAL80* gene therefore constitutively activates Gal4 and *GAL* gene expression^28^.

**Fig. 4:**
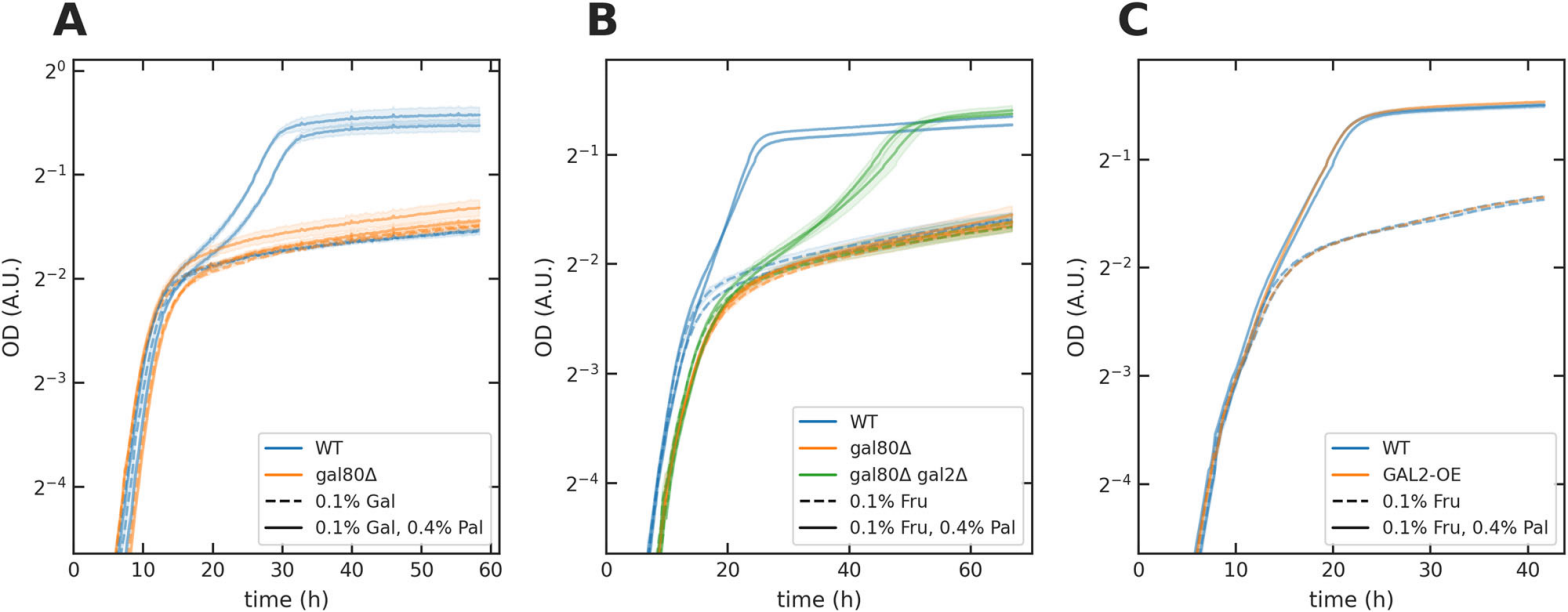
The Gal4 signal limits the use of palatinose. Using dashed lines to indicate single sugars and full lines to indicate mixtures, each curve represents one biological replicate; the shading shows the standard deviation of two technical replicates. **A** Compared to the wild-type, deleting *GAL80* limited growth in palatinose in galactose-palatinose mixture. **B** Deleting *GAL80* limited growth in palatinose in fructose-palatinose mixtures, which was partially alleviated by additionally deleting *GAL2*. **C** Over-expressing *GAL2* with the *CCW12* promoter (*GAL2-OE*) did not affect growth.

We observed that the *gal80Δ* strain either did not use or delayed using palatinose in both galactose- and fructose-palatinose mixtures (Fig. 4A, B). Focusing on the fructose-palatinose mixture where it is only the *GAL80* deletion that activates the *GAL* regulon, this delay vanished in a *gal80Δgal4Δ* mutant (Fig. S6A). Active Gal4 therefore likely prevented cells using palatinose.

Gal4 induces the genes *GAL1*, *GAL7*, and *GAL10*^29^, and this expression could deplete intracellular resources, such as ATP and amino acids, preventing *gal80Δ* cells from expressing the *MAL* regulon in palatinose mixtures. Deleting the entire *GAL1-10-7* locus in the *gal80Δ* mutant, however, did not change its phenotype (Fig. S6B); intracellular resources are unlikely to be limiting.

Gal4 also induces expression of *GAL2*, which encodes galactose permease, a hexose transporter. Surprisingly, we found that deleting *GAL2* did allow the *gal80Δ* cells at least partially to consume palatinose (Fig. 4B), implying that Gal2 might hinder growth in palatinose. Over-expressing *GAL2* in otherwise wild-type cells leads to transcript levels similar to the *gal80Δ* mutant (Fig. S6C). This mutant, however, had no obvious phenotype (Fig. 4C).

Active Gal4 and *GAL2* therefore together impede cells from metabolising palatinose.

### Active Gal4 prevents *MAL11* induction

We next used RNA-seq to determine how a constitutively active Gal4 in the *gal80Δ* mutant alters gene expression. We again chose to pair fructose with palatinose. To compare expression with and without active Gal4, we cannot use galactose, because it would activate Gal4 in the wild-type control, or glucose, because it would repress *GAL4* irrespective of Gal80’s presence^30^. Fructose however does not (Fig. S7A). With fructose, we know too that palatinose causes expression of the *MAL* regulon (Fig. 3A). We selected a fructose concentration that made the growth of the wild-type and *gal80Δ* strains as similar as possible to reduce confounding transcriptional changes generated by differing growth rates^31^. Both have an exponential growth rate of 0.36 h^−1^.

**Fig. 5:**
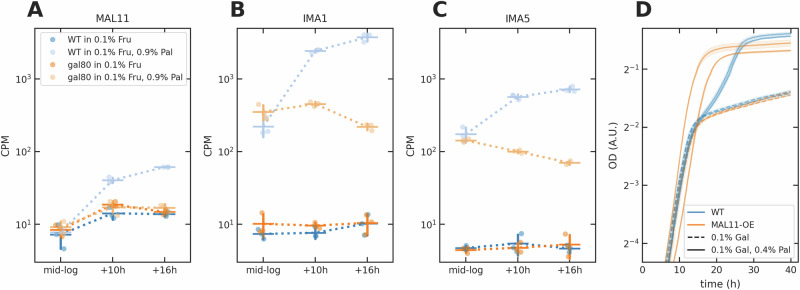
The Gal4 signal inhibits *MAL11* expression in palatinose. **A–C** The count per million reads (CPM) of *MAL11* (**A**), *IMA1* (**B**) and *IMA5* (**C)** transcripts. Data are shown as mean  ± standard deviation of three biological replicates (dots). **D** Over-expressing *MAL11* with the *CCW12* promoter (*MAL11-OE*) in the wild-type abolished the diauxie phenotype. We use dashed lines to indicate single sugars, full lines to indicate mixtures, and each curve represents one biological replicate; the shading shows the standard deviation of two technical replicates.

The *gal80Δ* deletion reduced the expression of the two isomaltase genes and the palatinose transporter, *MAL11* (Fig. 5A–C). With palatinose (lighter colours), the transcripts of the isomaltases in both the wild-type (blue) and the *gal80Δ* (orange) strains increased by the mid-log time point, but those of the mutant stabilised while the wild-type’s kept increasing (Fig. 5B, C). In contrast, the mutant’s *MAL11* gene was never induced, unlike the wild-type’s (Fig. 5A).

These results are consistent with active Gal4 repressing *MAL11*, either directly or indirectly, and so weakening the positive feedback in the *MAL* regulon. More intracellular palatinose activates *MAL11* expression via the palatinose-sensing *MAL* transcriptional regulators and so gives rise to more Mal11 transporters and so still more intracellular palatinose. With the low levels of Mal11 caused by active Gal4, however, we suspected that the mutant cells in a frucose-palatinose mixture imported enough palatinose to induce the isomaltase genes, but not enough to strongly induce *MAL11*’s expression. To test this hypothesis, we over-expressed *MAL11* in both the wild-type and the *gal80Δ* strains and reexamined the diauxie in a galactose-palatinose mixture. Both the diauxie in the wild-type (Fig. 5D) and the limited growth of the deletion mutant vanished (Fig. S8A), consistent with a *MAL* regulon that is more easily induced because of the higher intracellular palatinose concentrations generated by more Mal11.

### Mathematical modelling predicts that reduced *IMA* expression may abolish the galactose-palatinose diauxie

For the *MAL* regulon to activate the positive feedback through Mal11, the intracellular palatinose concentration should be high enough to drive *MAL11* transcription. We realised, however, that the isomaltases might prevent cells from reaching this strongly expressing state: if induced sufficiently early, the isomaltases may outcompete the regulon’s transcriptional activators for palatinose and weaken positive feedback by cleaving palatinose into fructose and glucose. A similar phenomenon may explain behaviour reported for maltose metabolism. There, cells over-expressing the maltase gene *MAL12* had a long lag in growth when switched from glucose to maltose^32^, likely because the high levels of Mal12 prevented cells inducing the genes for maltose transporters. In our RNA-seq results, Gal4 inhibits the expression of *MAL11* but not the isomaltases *IMA1* and *IMA5* (Fig. 5A–C). We therefore wondered how important the expression level of the isomaltases might be for galactose-palatinose diauxie.

**Fig. 6:**
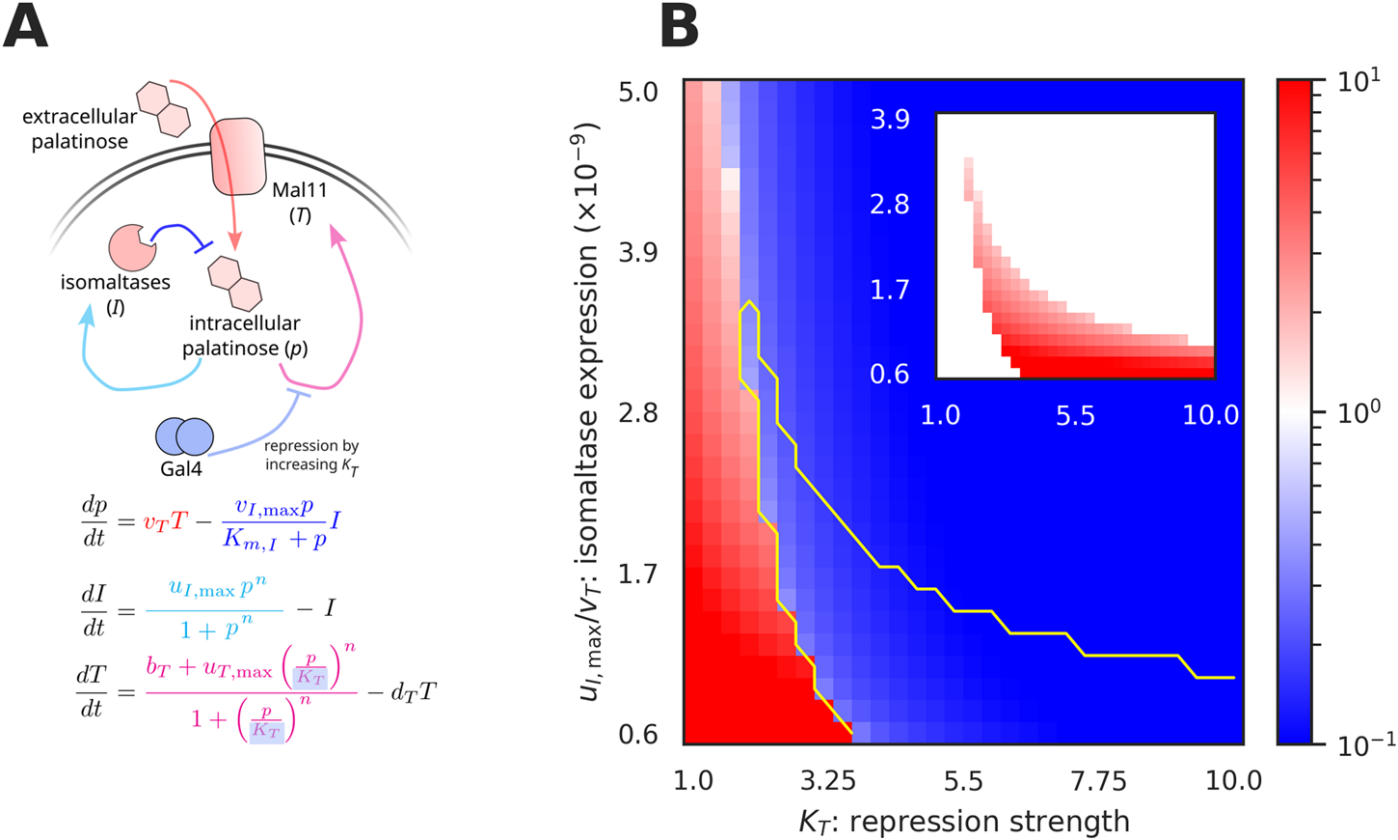
Mathematical modelling shows that galactose-palatinose diauxie depends on the levels of the isomaltases. **A** Three ordinary differential equations model the *MAL* regulon. The state variables are the concentrations of intracellular palatinose (*p*), isomaltases (*I*) and Mal11 transporter (*T*). The colours of the arrows match the corresponding terms in the equations. The presence of extracellular galactose increases *K*_*T*_. **B** The steady-state *p*/*K*_*T*_ value (colour bar) as a function of the repression strength, *K*_*T*_, and the ratio of maximal *IMA* expression to the palatinose import rate, ${u}_{I,\max }/{v}_{T}$. We define the system to be ON if *p*/*K*_*T*_ > 1 (red). The yellow contour marks a bistable region; the inset shows the value of *p*/*K*_*T*_ at the high steady state and the main figure the value at the low steady state. The wild-type strain has diauxie: it has a low *K*_*T*_ and is ON (red) in the presence of extracellular palatinose; adding extracellular galactose increases *K*_*T*_ sufficiently for the strain to be OFF. Parameter values are in Table S6; Hill number *n* = 3. The ratio ${u}_{I,\max }/{v}_{T}$ has a minimal value to prevent any steady states with infinite *p*, which we presume evolution avoids.

We first built a mathematical model of the *MAL* regulon with both positive and negative feedback (Fig. 6A): *MAL11* expression activates the transcriptional regulators by raising intracellular palatinose; *IMA1* and *IMA5* expression deactivates the regulators by lowering intracellular palatinose. We reduced the system to three variables by assuming that the activators rapidly dimerise and rapidly bind palatinose (Supplementary Note 1). Defining *p* as intracellular palatinose, *I* as the total levels of Ima1 and Ima5 together, and *T* as the levels of Mal11, we have three differential equations (Fig. 6A). We approximated isomaltase activity using the Michaelis-Menten equation^33^ and used Hill functions, modified with a basal rate *b*_*T*_ for *MAL11*, to describe gene expression. For simplicity, we used the degradation rate of *I* as the time scale and measured concentrations in units of the EC_50_ value, *K*_*I*_, of the Hill function for *IMA* expression. We imposed *K*_*T*_ > *K*_*I*_, so that cells express *IMA1* and *IMA5* before *MAL11* (Fig. 5A–C). The rate *d*_*T*_ models Mal11’s degradation, and *v*_*T*_ is the palatinose import rate, which increases with extracellular palatinose. We consider the regulon, and cells, to be ON if the steady-state intracellular palatinose *p* is greater than *K*_*T*_: then there is sufficient *p* to induce *MAL11* expression, increasing palatinose import and strengthening the positive feedback. Our model is similar to a recognised network motif with coupled positive and negative feedback^34,35^, although we assume that the negative feedback occurs for lower values of *p* than the positive feedback because *K*_*T*_ > *K*_*I*_.

We performed bifurcation analysis on two parameters: the EC_50_ of *MAL11* expression, *K*_*T*_, and the ratio of the maximal expression rate of the isomaltases to the palatinose import rate, ${u}_{I,\max }/{v}_{T}$. We considered a Hill number *n* = 3 (Fig. 6B), as well as *n* = 2 and *n* = 4 (Fig. S9). This analysis revealed three regions (Figs. 6B, S9): an ON region (red); an OFF region, where *p* < *K*_*T*_ (blue); and a bistable region, where one stable solution is OFF and the other ON.

To model galactose-palatinose diauxie, we let galactose repress *MAL11* by increasing *K*_*T*_, assuming that an unknown *GAL*-induced repressor competitively binds to the *MAL11* promoter (Fig. 6A). Without extracellular galactose but with palatinose, the wild-type strain is ON with a small *K*_*T*_ (Fig. 6B left). With extracellular galactose, the cells are OFF because of the *GAL*-increased *K*_*T*_ (Fig. 6B right). Depending on ${u}_{I,\max }/{v}_{T}$, the larger *K*_*T*_ causes cells to be either OFF or in the bistable region. Even when bistable, however, they remain OFF, being locked in the low steady state because of hysteresis: in the absence of extracellular palatinose, intracellular palatinose is low, and cells approach the bistable region from low steady-state *p*. The wild-type cells therefore exhibit diauxie, being OFF when extracellular galactose increases *K*_*T*_.

Our modelling also predicted that if the *GAL*-induced increase in *K*_*T*_ is not too large (*K*_*T*_ ≤ 3.4), a mutant strain with decreased *IMA* expression, and so smaller ${u}_{I,\max }/{v}_{T}$, may become ON despite the *GAL*-increased *K*_*T*_ (moving vertically from the blue to the red region in Fig. 6B). This strain should not have galactose-palatinose diauxie. The loss of one copy of the *IMA* genes might generate such a mutant.

### Loss of *IMA1* abolishes the galactose-palatinose diauxie

To test the model’s predicted sensitivity of diauxie, we deleted one of the *IMA* genes, which we expected might switch ON the *MAL* regulon even in the presence of both extracellular galactose and palatinose.

**Fig. 7:**
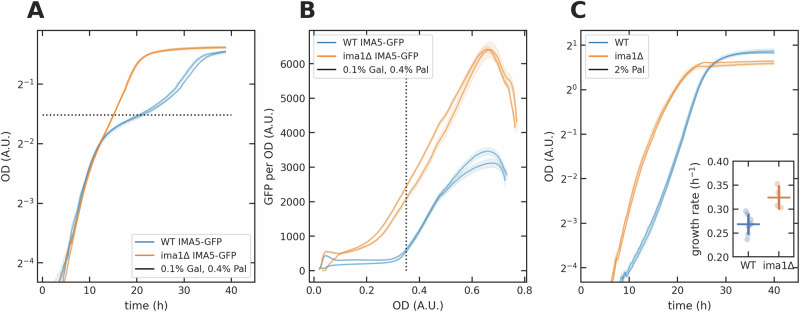
The preference of galactose over palatinose results from both repression by *GAL* and early expression of the isomaltases. In each panel, each curve represents one biological replicate; the shading shows the standard deviation of two technical replicates. **A, B** Deleting *IMA1* from the wild-type strain abolished diauxie in galactose-palatinose mixtures. The black dotted line marks the OD at which galactose was almost exhausted. **C** Deleting *IMA1* decreased the lag and increased the growth rate in 2% palatinose (*p* = 1.4 × 10^−3^ using an independent samples t-test); see inset: data are mean  ± standard deviation of at least four biological replicates.

We found that *ima1Δ* cells did lose diauxie in galactose-palatinose mixtures (Fig. 7A, B). Although *ima5Δ* cells did not (Fig. S8C), this behaviour remains consistent with isomaltase-induced negative feedback because deleting *IMA1* likely decreased isomaltase concentrations more than deleting *IMA5*: in palatinose, *IMA1*’s transcript levels are five-fold higher than *IMA5*’s (Fig. 5B, C) and their products, Ima1 and Ima5 proteins, have a similar *k*_cat_ and *K*_*M*_^33^. Without *IMA1*, cells likely have such low levels of isomaltase that the intracellular palatinose concentration is high enough to generate positive feedback even with the *GAL*-repressed *MAL11*. Supporting this interpretation, we found that a *gal80Δima1Δ* strain in galactose-palatinose mixtures lost the limited growth of the *gal80Δ* strain (Fig. S8B) and that deleting *IMA1* decreased the lag and increased the growth rate in palatinose compared to the wild-type strain (Fig. 7C), likely because of greater palatinose import through a more easily activated *MAL* regulon.

Our results show that losing one of the isomaltase genes, *IMA1*, abolishes the galactose-palatinose diauxie, and indicate that the negative feedback through the isomaltases can control diauxie, rather than the positive feedback seen in other systems^36^.

## Discussion

We have shown that budding yeast prioritises sugars other than glucose, consuming galactose before palatinose. This finding supports early foundational work that suggested galactose might be preferred over maltose^37^. We demonstrated that cells actively impose their preference, partly through the transcriptional regulator Gal4.

Our findings challenge current understanding. Although they are consistent with the observation that cells undergoing diauxie prioritise the carbon source allowing faster growth^38^, they are inconsistent with its converse. Both fructose and sucrose enable faster growth than palatinose (Fig. 1B), and faster growth than galactose, yet we observe no hallmarks of diauxie for cells growing in mixtures of either sugar with palatinose (Fig. 2A, B). Our results suggest further that cells prioritise carbon sources neither passively through dilution nor by a flux-sensing mechanism alone because faster growth typically implies a faster glycolytic flux, at least in different glucose concentrations^39^.

Cells likely combine a general flux-sensing mechanism, perhaps through the SNF1 kinase complex, yeast’s AMP kinase, and protein kinase A^40^, with targeted regulation specific to particular carbon sources. There is some evidence of this targeted regulation despite it being little studied. Both galactose^41^ and fructose^42^ repress the *SUC2* gene, which encodes for the invertase enzyme used to metabolise sucrose and raffinose. Galactose also represses *CYB2*^43^, an oxidoreductase used to metabolise lactate.

It is difficult to understand the origin of the different cellular behaviours in either glucose, fructose, or sucrose with palatinose. All three sugars allow similar growth rates (Fig. 1B), yet for these sugars we saw classic diauxie only for glucose-palatinose mixtures (Fig. 2A, B). For some concentrations of fructose and palatinose and less often for sucrose and palatinose, we observed a ‘shoulder’ in the growth rate over time (Fig. 2B, Fig. S5). Given that the OD in the mixtures is often initially indistinguishable from the OD on fructose or sucrose alone (Figs. 4B wild-type data & S5) and that cells immediately express the isomaltases in both mixtures (Figs. 3A & S5), these enzymes may at first be partially inactivated, perhaps allosterically or in some other way. Cells would then generate the growth-rate shoulder when they re-activate the enzymes, presumably when the fructose or sucrose concentration drops sufficiently. A similar phenomenon occurs in glucose-galactose mixtures where some cells express *GAL* enzymes but do not consume galactose^44^.

How cells might mechanistically recognise the different sugars and distinguish a higher concentration of one from a lower concentration of another is also puzzling because all enter glycolysis. One possibility is through extracellular sensing. The hexose sensors Snf3 and Rgt2 likely have different affinities for different hexoses^45^ and the G-protein coupled receptor Gpr1 favours sucrose over glucose^46,47^. Another possible mechanism is through the glycolytic enzyme hexose kinase 2, whose phosphorylation state changes in different carbon sources^48^.

**Fig. 8:**
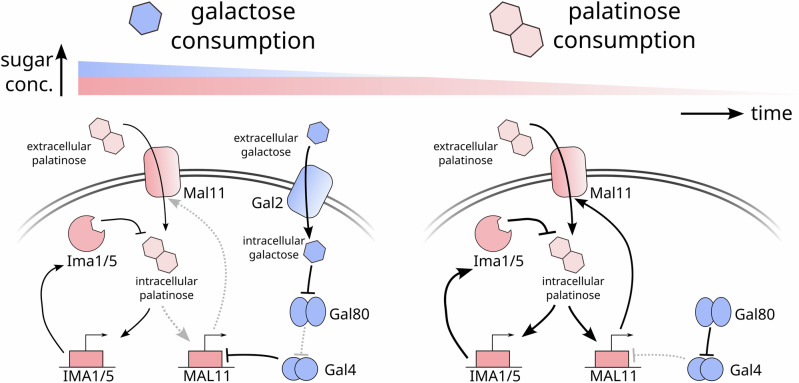
Cells use an active, specific mechanism to implement galactose-palatinose diauxie. Initially they consume galactose (left). Active Gal4 indirectly represses *MAL11*. This repression together with the negative feedback through Ima1 and Ima5 reduces intracellular palatinose concentrations, weakening positive feedback in the *MAL* regulon (greyed out arrows). When galactose runs out (right), Gal80 inactivates Gal4. The repression on *MAL11* lifts, and the higher levels of the Mal11 transporters increase intracellular palatinose, further activating *MAL11*. Positive feedback in the *MAL* regulon strengthens and becomes self-reinforcing, and cells consume palatinose.

Our results suggest that galactose prevents palatinose metabolism by inhibiting positive feedback in the *MAL* regulon (Fig. 8). As cells consume galactose, they activate Gal4 and repress *MAL11*, the palatinose transporter. This repression together with early expression of the isomaltases, *IMA1* and *IMA5*, prevents intracellular palatinose reaching sufficient concentrations to induce higher expression of *MAL11*. As cells exhaust galactose, however, Gal80 inactivates Gal4, and Gal4’s repression of *MAL11* lifts, import of palatinose increases, and positive feedback strengthens.

Prioritising activation of the isomaltase genes may have been selected to prevent excessive intracellular palatinose. Maltose, another substrate of the *MAL* regulon, is toxic at high intracellular concentrations and inhibits translation^49^. Its import, like palatinose’s, uses the proton-motive force and so may be deleterious through draining cellular energy^50^.

The *MAL* regulon’s characteristics allow flexibility in the decision-making: we showed that the loss of the *IMA1* gene abolishes diauxie. *IMA1*, like most *MAL* genes, is near the telomeres, where gene loss and duplication are common^17^. On only short evolutionary timescales cells might therefore be able to switch between co- and sequential consumption.

We do not know how active Gal4 represses *MAL11*. Although Gal4 is reported to directly regulate only 12 genes^51,52^, our transcriptomic data imply that it affects the expression of a larger set, including the hexose transporters and genes controlling ribosome biogenesis (Fig. S10C & D), as well as the *GAL* regulon and other known non-*GAL* targets^51–53^. None of these genes, however, are transcription factors whose expression Gal4 could promote to repress *MAL11*, perhaps suggesting that it is Gal4 itself that represses.

A puzzling discovery is that deleting *GAL2*, the gene for galactose permease, partly alleviated the effects of constitutively activating Gal4, allowing *gal80Δ* cells to re-consume palatinose in galactose-palatinose mixtures (Fig. 4B). Similarly, slow growth of a *gal80Δ* mutant in raffinose is partly lifted by deleting *GAL2*^54^. Perhaps removing *GAL2* affects expression of nearby non-coding RNAs in the genome, such as the overlapping ncRNA *SUT692*^55^, although its function is still unknown.

Our results suggest that budding yeast’s preference for glucose is not unique and that cells actively regulate to enforce preferences for other sugars, such as galactose. We do not understand why cells prioritise galactose and glucose over palatinose more than they do fructose or sucrose despite cells growing faster on fructose and sucrose than they do on galactose. This behaviour suggests that cells do more than maximise growth rates, even in laboratory conditions. Active regulation is presumably necessary because of intracellular constraints^56^, but why these constraints should become alleviated in fructose and sucrose is unclear. Perhaps some of the behaviours we see are under only weak selection because yeast rarely encounter the corresponding combinations of sugars in the wild. More generic regulatory mechanisms may then suffice^57^, such as control by SNF1 kinase and the repressor Mig1^11^. Alternatively, for some sugars, competition may be fiercer than others, and so cells prioritise these sugars in an effort to starve competing organisms rather than for the sugars’ intrinsic values — such strategies can be evolutionarily stable^58^.

Taken together, our findings imply that carbon-sensing is too important for cells to regulate with only generic mechanisms and highlight the need both to delineate the decision-making strategies used and to determine how they are conserved across different species.

## Methods

### Strains and growth media

We list strains and constituents of the media used in Supplementary Tables 1 and 2. The BY4741-background strains are auxotrophic and the FY4-background strains are prototrophic^59^. Strains were pre-cultured in synthetic complete (SC) media supplemented with 2% (w/v) sodium pyruvate for two days before experiments, unless specified otherwise (Supplementary Experimental Methods 1.1). Pyruvate is a gluconeogenic carbon source and ensures cells have the same minimal glycolytic activity before we add any sugars. We then diluted cultures six-fold six hours before an experiment with fresh SC media with 2% (w/v) sodium pyruvate to ensure cells are at exponential growth when the experiment begins. During an experiment, we grew auxotrophic strains in SC or LoFlo media and prototrophic strains in minimal media (Delft media)^60,61^, both supplemented with carbon sources.

### Creating yeast strains

We followed a standard protocol using lithium acetate and polyethylene glycol (PEG) to transform yeast^62^. Transformants were confirmed by colony PCR and Sanger sequencing (MRC Protein Phosphorylation and Ubiquintylation Unit, Dundee). We list all plasmids in Supplementary Table 3 and all oligos in Supplementary Data 1. See also Supplementary Experimental Methods 1.2 for multiplex CRISPR, which we used to delete the *GAL1-10-7* locus.

### Growth assay in plate readers

We used plate readers (Tecan, Infinite M200 Pro or F200) to measure the dynamics of growth and fluorescence (Fig. 1A). Cells were grown in SC + 2% (w/v) sodium pyruvate in a 30 °C shaking incubator at 180 rpm for about 40 hours and then diluted by six-fold 6–8 hours before the experiment. Before harvesting, we added 20 *μ*L 10x sugar stock or water to each well, and cultures of each strain were then centrifuged at 3500 rpm for 3 minutes and the supernatant removed. We washed cells using the appropriate media base once for experiments with SC or LoFlo and twice for experiments with Delft media. Cells were then re-suspended so that the initial OD was below 0.2 as measured by a spectrophotometer. Finally, we added 180 μL re-suspended culture to each well to give a final volume of 200 μL. We then moved the 96-well plate into the plate reader at 30 °C with linear shaking at an amplitude of 6 mm and measurements taken every 10 minutes.

The plate-reader data are typically time series of 96 wells with both OD and fluorescence readings. We used a Python package, omniplate (version 0.9.95)^20^, to analyse the data. Our typical pipeline is: (1) ignore any contaminated wells; (2) average over technical replicates and calculate the standard deviation; (3) subtract the OD and fluorescence background of the media; (4) correct the non-linearity between OD and the cell number when OD is high^21^; (5) estimate the specific growth rate ($d/dt\,\log OD$) using a Gaussian process^23^, along with other quantities such as maximal OD; (6) if fluorescence is measured, correct the auto-fluorescence using untagged cells and spectral unmixing^22^; (7) calculate the fluorescence reading per OD.

### Measuring sugar concentrations by GC-MS

#### Growing the cells

We grew cells of the FY4 wild-type strain in SC + 2% (w/v) sodium pyruvate in a 30 °C shaking incubator at 180 rpm for about 40 hours and then diluted by six-fold six hours before the experiment. When the experiment began, we washed the cells twice with Delft media without carbon sources and then inoculated into 250 mL flasks with 25 mL Delft media supplemented with the desired concentrations of galactose and palatinose. The volume of inoculated cells was calculated to make the initial OD 0.05, and then we topped up the volume of each culture to 26 mL. The cultures were then incubated in a 30 °C shaking incubator at 180 rpm.

#### Harvesting the spent media

To harvest the spent media, we sampled 1 mL of each culture into a 15 mL Falcon tube placed on ice and then immediately put the flasks back into the shaking incubator to minimise the impact of sampling. From each 1 mL sample, we transferred 2 × 200 μL samples into two wells of a 96-well microplate for OD measurement in a Tecan plate reader (Tecan, Infinite M200 Pro). The remaining volumes in the samples were centrifuged at 4000 rpm for 15 minutes at 4 °C, and then we transferred 50 μL of the supernatant into a GC vial and stored at -20 °C. We harvested samples at 0, 10, 15, 20, 25, 30, 40, 50, 60, 70 and 80 h and measured the final OD at 90 h. In parallel, we measured with the same plate reader the OD of cultures in 0.1% galactose as a negative control.

#### Sample and standards preparation for sugar analysis

To the 50 μL spent media, we added 5 μL of the internal standard (3 mg/mL myristic acid d27 dissolved in water: methanol: isopropanol in a ratio of 2: 5: 2, v/v/v). The contents of the GC vial were evaporated to dryness in a Gene-Vac EZ-2 Elite evaporator, and trimethylsilylated with 50 μL pyridine: N-methyl-N-trimethylsilyltrifluoroacetamide (1:4) for gas chromatography quadrupole time-of-flight mass spectrometry (GC/QTOF-MS) analysis of the sugars.

#### Gas Chromatography-Mass Spectrometry (GC-MS) analysis

The sugar concentrations were analysed on an Agilent 7890B gas chromatogram (GC) coupled to an Agilent 7200B quadrupole time-of-flight mass spectrometer (QTOF-MS) with GERSTEL multipurpose sampler (MPS) robotics (Anatune). Trimethysilylated samples (1 μL) were injected at a split ratio of 10:1, with a split flow of 10 mL/min into a DB-5ms 40 m  × 250 *μ*m  × 0.25 *μ*m GC column (Agilent Technologies). We used helium as the carrier gas at a flow rate of 1 mL/min and set the inlet to 250 °C and programmed the GC oven to 60 °C for 1 min, followed by ramping at 10 °C/min to 325 °C, where it was held for 10 min. The ion source was set to 230 °C, 35 μA filament current, 70 eV electron energy, and we scanned the mass range of 60–900 m/z at an acquisition rate of 4 spectra/s with a solvent delay of 5 min. Total ion chromatograms and mass spectra were analysed using the Agilent MassHunter Qualitative Analysis B.10.00 software, and peak areas calculated using the Agile 2 integrator method.

### RNA measurements

#### Growing and harvesting cells

We harvested approximately four OD units of cells, by sampling *x* mL of each culture, such that the value of *OD* ⋅ *x* is around 4, and then centrifuging the cells at 3500 rpm for 3 minutes at 4 °C. The supernatant was removed and the cell pellets stored in -80 °C if RNA extraction did not immediately follow.

#### Extracting RNA

We adapted a column-based protocol in^63^ to extract RNA. We thawed the cell pellets on ice and then resuspended with 400 *μ*L RNA binding buffer (Zymo, #R1013-2). The mixtures were then transferred to 2 mL screw cap tubes with zirconia beads inside, and then cell lysis performed using the PreCellys Evolution homogeniser (Bertin Instruments)—the samples were shaken at 6000 rpm for 10 seconds for three cycles, with a 10-second pause between each cycle, before being placed on ice for one minute. We repeated the shaking-ice bath process five further times. Then we centrifuged the lysates for 90 seconds and transferred each supernatant to a Zymo Spin IIICG column (Zymo, #C1006) and centrifuged again. We then mixed the flow through with 400 *μ*L 100% ethanol, transferred to a Zymo Spin IIC column (Zymo, #C1011), and centrifuged at 12000 × *g* for one minute. With the RNA being on the column, we discarded the flow through. We then sequentially added and centrifuged through the column 400 *μ*L DNA/RNA prep buffer (Zymo, #D7010-2), 600 *μ*L DNA/RNA wash buffer (Zymo, #D7010-3), and 400 *μ*L DNA/RNA wash buffer, discarding all flow through. Finally, we centrifuged the column again before adding 30 μL nuclease free water (Ambion, #AM9937) to elute the RNA. All steps of centrifugation were performed at 12000 × *g* for one minute unless otherwise specified.

We measured the RNA concentrations with a spectrophotometer (DeNovix, #DS-11) and confirmed the quality of the RNA samples using a Fragment Analyzer (Advanced Analytical Technologies, Inc.) with the Standard Sensitivity RNA Analysis Kit (Agilent, #DNF-471).

#### RNA-seq experiment

We grew cells of the wild-type FY4 and *gal80Δ* strains in SC + 2% (w/v) sodium pyruvate in a 30 °C shaking incubator at 180 rpm for about 40 hours and then diluted by six-fold six hours before the experiment began. Next the cells were washed twice with Delft media without carbon sources and then inoculated into 250 mL flasks with 25 mL Delft media supplemented with the desired concentrations of fructose and palatinose. We calculated the volume of inoculated cells to make an initial OD of 0.005 and topped up the volume of each culture to 26 mL. The cultures were incubated in a 30 °C shaking incubator at 180 rpm.

We harvested samples at three time points: mid-log (at OD 0.3), 10 hours after mid-log, and 16 hours after mid-log (Fig. S7B).

Edinburgh Clinical Research Facility performed quality control, library preparation, and sequencing. They used a Fragment Analyser Automated Capillary Electrophoresis System (Agilent Technologies Inc, #5300) with the Standard Sensitivity RNA Analysis Kit (#DNF-471-0500) for quality control and an Qubit 2.0 Fluorometer (Thermo Fisher Scientific Inc, #Q32866) with the Qubit RNA broad range assay kit (#10210) for quantification. To quantify DNA contamination, an Qubit dsDNA HS assay kit (#Q32854) was used.

They generated libraries from 400 ng of each total RNA sample with the QuantSeq 3’ mRNA Library Prep Kit REV for Illumina (Lexogen Inc, #016) according to the manufacturer’s protocol. These libraries were then quantified by fluorometry with the Qubit dsDNA High Sensitivity assay and assessed for quality and fragment size with the Agilent Fragment Analyser with the SS NGS Fragment 1–6000 bp kit (#DNF-473-33).

They performed 2 × 50 bp paired-end sequencing on the NextSeq 2000 platform (Illumina Inc, #20038897) using NextSeq 1000/2000 P2 Reagents (100 cycles) v3 (#20046811), which produced 46.49 Gbp data. The data produced by the NextSeq 1000/2000 Control Software (Version 1.4.1.39716) was then automatically uploaded to BaseSpace (Illumina) and converted into FASTQ files.

We carried out RNA-seq alignment and quality control following Haynes et al.^64^ using code written in Nextflow^65^ (Fig. S7C--E) and available in a git repository: https://github.com/DimmestP/nextflow_paired_reads_pipeline. We list the software versions we used in Supplementary Table 4. We adapted the genome annotation file from the longest transcripts taken from Table S3 in^66^, and for genes without an reported 3’UTR in^66^, we assigned a default-length UTR of 125 nt as the median length is reported at 128 nt. We modified the annotations of some *MAL* genes—*MAL32*, *IMA1*, *MAL11*, and *MAL12*—and some genes neighbouring a *MAL* gene—*VTH1*, *HXT8*, *VTH2*, and *ALR2*—according to their actual 3’ ends from the reads in our experiment. We also added the annotation of *ZNF1* (*YFL052W*), which was missing. The output of this pipeline is a 5697 × 36 table with raw counts, which we used for differential expression analysis with DESeq2 (version 1.34.0)^67^. We then defined the set of differentially expressed genes between two conditions by $| {\log }_{2}{{\rm{fold\,change}}}| > 0.5$ and the adjusted p-value  < 0.05 for all three time points (Fig. S10A & B & Fig. S11). Both the adjusted p-value and the ${\log }_{2}{{\rm{fold\,change}}}$ were calculated with DESeq2^67^.

### Statistics and reproducibility

To infer the specific growth rate from OD measurements, we used a Gaussian process^23^; to compare specific growth rates between different conditions, we used an independent samples t-test.

For replicates in plate-reader assays, we had two technical replicates of two biological replicates for each strain and medium tested.

For the GC-MS and RNA-seq experiments, we had three biological replicates.

### Reporting summary

Further information on research design is available in the Nature Portfolio Reporting Summary linked to this article.

## Supplementary information

Supplementary Information

Description of Additional Supplementary Files

Supplementary Data 1

Reporting summary

## Data Availability

All plate-reader data are at 10.7488/ds/7685; the RNA-seq data are on the Gene Expression Omnibus (GEO) of the National Center of Biotechnology Information (NCBI) with accession number GSE240743; the data underlying all figures is at 10.5281/zenodo.14840342.

## Code availability

All computer code to generate the figures is at 10.5281/zenodo.14865247.
