# Supplementary material for: The type of carbon source not the growth rate it supports can determine diauxie in Saccharomyces cerevisiae
Source: Commun Biol. 2025 Feb 27;8:325. doi: 10.1038/s42003-025-07747-z (PMC11868555; doi:10.1038/s42003-025-07747-z)
Supplement: Supplementary file 1 — Supplementary Information [file 42003_2025_7747_MOESM1_ESM.pdf]

Supplementary: The type of carbon source not the growth rate it supports can determine diauxie in *Saccharomyces cerevisiae*

Y Huo, W Danecka, I Farquhar, K Mailliet, T Moses, EWJ Wallace, PS Swain.

1 Supplementary Experimental Methods

1.1 Quantifying growth dynamics for cells with non-pyruvate growth history

Strains were pre-cultured in synthetic complete (SC) media supplemented with 2% (w/v) glucose or raffinose overnight or with palatinose for two days, so the cultures are at late exponential phase. We then diluted cultures six-fold six hours before an experiment begins with fresh SC media supplemented with 2% (w/v) corresponding sugar, to ensure that the cultures are at exponential phase when the experiment starts. The steps after follow the Methods section in the main text.

1.2 Multiplex CRISPR for deleting the *GAL1-10-7* locus

The length of the *GAL1-10-7* locus is 6181 bp. If we use standard techniques to delete this locus, the efficiency will be low because the two 40-bp homology arms are far from each other. Instead we therefore used the multiplex CRISPR technique to introduce a markerless deletion of the *GAL1-10-7* locus.

Our design was to induce one double-strand break near each end of the *GAL1-10-7* locus with the Cas9 protein led by a guide RNA. Each break should trigger homology directed repair using the homologous recombination template we added, which contains the desired genomic edits. We obtained the Cas9 cassette, together with a *NAT* marker, by PCR from the Amp1284 plasmid and separately incorporated the double-stranded guide DNAs into the Amp1278 plasmid by *Esp3I* assembly. We obtained the desired region on each plasmid using PCR. The homologous recombination template has an upstream and a downstream homology arm, each 500 bp. To obtain

the template, we used PCR to amplify the upstream and downstream homology arms separately and then joined them via Gibson assembly. We removed the protospacer adjacent motifs (PAMs), targeted by the guide RNAs, from the template by introducing a point mutation on the PCR primer.

The three components were then transformed into yeast cells using the standard protocol [1]. We plated the cells on a XY-glucose plate with nourseothricin to select cells with the Cas9 cassette. After two days, the transformants were re-inoculated on a fresh XY-glucose plate as patches, because the homologous recombination template does not contain a selection marker. Once the patches of cells grew, we ran colony PCR and select the patch that had the strongest band for our edit. We streaked cells from that patch of cells into single colonies on a fresh XY-glucose plate and ran colony PCR again when that plate was ready. The process, which alternates between streaking and colony PCR, continued until the band for wild-type strains eventually disappeared. Finally, the colonies were confirmed again with Sanger sequencing.

1.3 Monitoring single-cell growth and gene expression with the ALCATRAS microfluidic device

We prepared the overnight cultures and diluted before experiments using the same method we used for the plate-reader experiments (see Methods in the main text). Cells were then loaded into the three-chamber version of the ALCATRAS microfluidic device [2, 3]. In the device, cells were exposed to 2% sodium pyruvate during the first four hours of imaging and then to either 1% palatinose, 1% palatinose and 0.2% galactose, or 1% palatinose and 0.2% sucrose.

1.4 Quantifying transcripts by RT-qPCR

Before the RT-qPCR experiment, we designed primer pairs that target the genes of interest using the PrimerQuest tool (Integrated DNA Technologies), with optimal T_m at 62 °C, optimal GC percentage at 50%, optimal primer size at 22 bp, and optimal amplicon length at 100 bp. Primer pairs for the reference genes *ALG9* and *ACT1* were taken from Barrass *et al.* [4]. We validated all primers by running an RT-qPCR experiment with the primer pairs and a serial dilution of input

cDNA (1x, 5x, 25x and 125x) — we consider a primer pair validated if the melting temperature of the product is unique and consistent across different dilution factors and if the C_q value decreases linearly with the logarithm of the dilution factor. We list all primers in Supplementary Data 1.

An RT-qPCR experiment comprises three steps: DNase treatment, reverse transcription, and quantitative PCR.

First, we treated the extracted RNA samples (1–3 μg each) with DNaseI (ThermoFisher, #EN0525) in the presence of RNase inhibitor (ThermoFisher, SuperAseIn) at 37 °C for 1 hour and then deactivated the DNaseI with DNase inactivation reagent (Invitrogen, #AM1907) at room temperature for five minutes before spinning down the DNase inactivation reagent.

Second, we added the random primer mix (NEB, #S1330S) to each sample, split the sample into two — each 5 μL for the +RT and -RT reactions, and denatured the RNA at 70 °C for 5 minutes, before moving the samples onto ice. Then we added into each +RT reaction, 0.75 μL 10 mM dNTP mix, 0.25 μL RNase inhibitor, 0.5 μL Superscript IV Reverse Transcriptase (Invitrogen, #18090010), 2 μL first strand synthesis buffer and 1.5 μL nuclease-free water. For the -RT reactions, we added the same reagents except replacing the transcriptase with nuclease-free water. Next the samples were incubated at 25 °C for five minutes and then 55 °C for one hour. The product (cDNA) was then diluted by about 20-fold with nuclease-free water.

Third, we ran the quantitative PCR with a 384-well microplate. Each well contained 1.6 μL 2x Brilliant III Ultra-Fast SYBR Green QPCR Master mix (Agilent, #600886), 0.4 μL primer pair mix (4 μM each), and 2 μL cDNA. We then moved the microplate into a LightCycler 480 machine (Roche), with *Taq* activation at 95 °C for three minutes, 40 cycles of amplification (95 °C for five seconds and 60 °C for 10 seconds with green fluorescence reading), and melt-curve measurement (ramping up from 65 °C to 95 °C at 0.29 °C/s).

The data from an RT-qPCR experiment are time series of fluorescence from each well. We used the manufacturer's software (Roche LightCycler 480 SW 1.5) to extract the C_q values of each curve: the number of cycles required to amplify the input cDNA to a fixed threshold. We used an R package, `tidyqpcr` (version 1.1), to analyse the C_q data [5]. Our pipeline had three steps: (1) averaging over technical replicates to find the mean C_q for each gene in each biological replicate; (2)

calculating ΔC_q for each gene in each biological replicate ny subtracting the mean C_q for each gene in each biological replicate by the mean of the mean C_q of all three reference genes; (3) calculating $\Delta\Delta C_q$ for each gene in each biological replicate by subtracting the ΔC_q value of that gene in each biological replicate by the mean ΔC_q of all biological replicates of that gene in the control group.

2 Supplementary Note 1: Mathematically modelling the response to palatinose

2.1 A deterministic description and finding steady states

The *MAL* regulon has five components: a transporter Mal11 (T), two isomaltases Ima1 (I_1) and Ima5 (I_2) and two activators Mal13 (R_1) and Znf1 (R_2) [6]. Its activation requires both activators, and we assume they function as a heterodimer (R_c), which becomes active (R_c^*) after binding intracellular palatinose (p).

We consider the following reactions: palatinose import (v_T); palatinose hydrolysis by the two isomaltases ($v_{I,1}$ and $v_{I,2}$); palatinose's association and dissociation with the activator heterodimer (k_p and k'_p); dimerisation between the two activators (k_R and k'_R); and the expression of each gene (u_{gene}). We assume that palatinose hydrolysis follows Michaelis-Menten kinetics, that association and dissociation reactions follow the law of mass action, and that the rate of gene expression is a Hill function of R_c^* with any basal expression, denoted as $u_{gene}(R_c^*)$. The only exceptions are $u_{R,1}$ and $u_{R,2}$, which we approximate as constant following our RNA-seq data (Fig. S7F). We do not consider dilution by growth for simplicity.

The complete model comprises eight ordinary differential equations:

$$\dot{p} = v_T T - v_{I,1}(p) I_1 - v_{I,2}(p) I_2 - k_p R_c p + k'_p R_c^* \quad (1)$$

$$\dot{T} = u_T(R_c^*) - d_T T \quad (2)$$

$$\dot{I}_1 = u_{I,1}(R_c^*) - d_{I,1} I_1 \quad (3)$$

$$\dot{I}_2 = u_{I,2}(R_c^*) - d_{I,2} I_2 \quad (4)$$

$$\dot{R}_1 = u_{R,1} - k_R R_1 R_2 + k'_R R_c - d_{R,1} R_1 \quad (5)$$

$$\dot{R}_2 = u_{R,2} - k_R R_1 R_2 + k'_R R_c - d_{R,2} R_2 \quad (6)$$

$$\dot{R}_c = k_R R_1 R_2 - k'_R R_c - k_p R_c p + k'_p R_c^* \quad (7)$$

$$\dot{R_c^*} = k_p R_c p - k'_p R_c^* \quad (8)$$

We further assume differences in time scales so that the association and dissociation between p and R_c and between R_1 and R_2 are at equilibrium and that levels of R_1 and R_2 are at quasi-steady state. Consequently,

$$k_p R_c p = k'_p R_c^* \tag{9}$$

$$k_R R_1 R_2 = k'_R R_c \tag{10}$$

$$u_{R,1} = d_{R,1} R_1 \tag{11}$$

$$u_{R,2} = d_{R,2} R_2 \tag{12}$$

which gives

$$R_c^* = \frac{k_p k_R u_{R,1} u_{R,2}}{k'_p k'_R d_{R,1} d_{R,2}}\, p \tag{13}$$

and we can write all rate of gene expression $u_{gene}(R_c^*)$ as functions of p.

The cleavage of palatinose by Ima1 and Ima5 follows Michaelis-Menten kinetics within the range of concentration we used, and their kinetic parameters are similar [7]. We therefore set $v_{E,1}(p) = v_{E,2}(p) \equiv v_E(p)$. Although cells degrade Ima5 faster than Ima1 at 37°C [7], the rates at 30°C are unreported, and so, again for simplicity, we use the same degradation rate for both: $d_{I,1} = d_{I,2} \equiv d_I$.

Further, we let the expression of the two isomaltases change in proportion with each other, so that $u_{E,1}(p) = \alpha u_{E,2}(p)$ for a constant α. Then defining $I \equiv I_1 + I_2$ and writing $u_I(p) = (1+\alpha) u_{I,1}(p)$, we have a simplified system of three equations:

$$\frac{dp}{dt} = v_T T - v_I(p) I \tag{14}$$

$$\frac{dT}{dt} = u_T(p) - d_T T \tag{15}$$

$$\frac{dI}{dt} = u_I(p) - d_I I \tag{16}$$

where

$$v_I(p) = \frac{v_{I,\max} p}{K_{m,I} + p} \tag{17}$$

$$u_T(p) = \frac{b_T + u_{T,\max}\left(\frac{p}{K_T}\right)^n}{1 + \left(\frac{p}{K_T}\right)^n} \tag{18}$$

$$u_I(p) = \frac{b_I + u_{I,\max}\left(\frac{p}{K_I}\right)^n}{1 + \left(\frac{p}{K_I}\right)^n} \tag{19}$$

For convenience, we show all parameters in Table S5.

Our model is similar to a recognised network motif with coupled positive and negative feedback [8, 9], but we will assume that the negative feedback is faster than the positive feedback (i.e., $K_I < K_T$).

2.2 Setting the parameters

Values for d_T, d_I, $K_{m,I}$, and $v_{I,\max}$ have been reported [10, 7]. For K_I and K_T, a previous measurement of intracellular maltose for a strain grown in maltose is 1.3 mM [11, p. 102], so we assume that K_I and K_T are of order 1 mM.

We also assume that the maximal production rate of *MAL11*, $u_{T,\max}$, is the same order of magnitude as the maximal production rate of *GAL1*, from Ref [12].

We consider Hill number $n = 2,\ 3$ and 4.

2.3 A non-dimensionalised description and the steady states

To simplify the analysis, we non-dimensionalise Eqs. 14–16 by normalising the concentrations by K_I and the time by d_I. By substituting the definition of the non-dimensionalised variables and

parameters in Table S6 into Eqs. 14–19, we have:

$$\frac{dp'}{dt'} = v'_T T' - \frac{v'_{I,\max} p'}{K'_{m,I} + p'} I' \tag{20}$$

$$\frac{dT'}{dt'} = \frac{b'_T + u'_{T,\max}\left(\frac{p'}{K'_T}\right)^n}{1 + \left(\frac{p'}{K'_T}\right)^n} - d'_T T' \tag{21}$$

$$\frac{dI'}{dt'} = \frac{b'_I + u'_{I,\max} p'^n}{1 + p'^n} - I' \tag{22}$$

We will omit the prime ($'$) symbol of each non-dimensionalised parameters and variables henceforth as well as in the main text and will focus on analysing the non-dimensionalised version of the model.

Solving at steady state, denoted by the asterisk (*), we have:

$$\begin{aligned} \frac{v_{I,\max} p^*}{K_{m,I} + p^*} &= v_T T^*/I^* \\ T^* &= \frac{b_T + u_{T,\max}\left(\frac{p^*}{K_T}\right)^n}{1 + \left(\frac{p^*}{K_T}\right)^n} \Bigg/ d_T \\ I^* &= \frac{b_I + u_{I,\max} p^{*n}}{1 + p^{*n}} \end{aligned} \tag{23}$$

or, combining,

$$\frac{v_{I,\max} p^*}{K_{m,I} + p^*} \times \frac{b_I + u_{I,\max} p^{*n}}{1 + p^{*n}} \times \frac{1 + \left(\frac{p^*}{K_T}\right)^n}{b_T + u_{T,\max}\left(\frac{p^*}{K_T}\right)^n} \times \frac{d_T}{v_T} - 1 = 0 \tag{24}$$

which simplifies into a polynomial equation of p, and the number of positive roots gives the number of steady states.

When $b_I = 0$, Eq. 24 can be re-written as:

$$\frac{v_{I,\max} p^*}{K_{m,I} + p^*} \times \frac{\alpha p^{*n}}{1 + p^{*n}} \times \frac{1 + \left(\frac{p^*}{K_T}\right)^n}{b_T + u_{T,\max}\left(\frac{p^*}{K_T}\right)^n} \times d_T - 1 = 0 \tag{25}$$

where $\alpha \equiv \frac{u_{I,\max}}{v_T}$, which is biologically the maximum expression rate of isomaltase normalised by the palatinose import rate.

The positive root(s) of Eq. 25 are the steady states. We regard a steady state with $p^* > K_T$ as ON because *MAL11* expression is then induced; otherwise a steady state is OFF.

2.4 Bifurcation analysis on K_T and α

We wish to understand how the isomaltase expression level and the repression strength by *GAL* affects the steady state of the *MAL* regulon and so generates or abolishes diauxie.

To model *GAL* repression, we let an unknown *MAL* repressor downstream of Gal4 compete with the activator at MAL11's promoter, making K_T increase when the GAL regulon is active. We model increasing isomaltase expression by increasing α. We numerically solve Eq. 25 for steady-state p^* for different K_T and α (Fig. 6B & S9).

From our RNA-seq data, *IMA1* and *IMA5* are expressed before *MAL11* (Fig. 5A–C), so we let $K_T > K_I$.

To determine the range of α, we note that the left hand side of Eq. 25 is bounded when $p^* \to +\infty$:

$$\lim_{p^* \to +\infty} \left(\frac{v_{I,\max} p^*}{K_{m,I} + p^*} \times \frac{\alpha p^{*n}}{1 + p^{*n}} \times \frac{1 + \left(\frac{p^*}{K_T}\right)^n}{b_T + u_{T,\max} \left(\frac{p^*}{K_T}\right)^n} \right) \times d_T - 1 = \frac{v_{I,\max} \alpha d_T}{u_{T,\max}} - 1 \tag{26}$$

To ensure a steady state exists for all K_T, we require this limit to be positive:

$$\frac{v_{I,\max} \alpha d_T}{u_{T,\max}} - 1 > 0 \tag{27}$$

which is

$$\alpha > \frac{u_{T,\max}}{v_{I,\max} d_T} \approx 5.9 \times 10^{-10} \tag{28}$$

from Table S5. Biologically this assumption means that we cannot have too little isomaltases expressed or too high a palatinose import rate, otherwise the cells will accumulate infinitely high amount palatinose, leading to cell death [11, pp. 90–116].

2.5 Alternative ways of modelling *GAL* repression

We think it unlikely that *GAL* repression decreases either v_T or $u_{T,\max}$, rather than increasing K_T, because the *gal80*Δ *ima1*Δ mutant, which has an active GAL regulon, grows in 2% palatinose with the same rate as the wild-type. Mathematically, if we assume that the growth rate λ is proportional to the palatinose import rate and that 2% palatinose is sufficient to fully activate the *MAL* regulon, then

$$\lambda \propto v_T T = v_T u_{T,\max}/d_T \tag{29}$$

at steady state from Eq. 23. Consequently, if λ is similar between the wild-type and double mutant strain, so too is the product $v_T u_{I,\max}$, implying by Occam's razor that neither has changed.

Supplementary Tables

Strain ID	Genotype	Parent	Reference
SL229	BY4741 (wildtype)		[13]
SL365	FY4 (wildtype)		[13]
SL1121	*IMA1-GFP*	SL229	
SL1360	*ima5*Δ	SL229	
SL1425	*ima1*Δ	SL229	
SL1489	*gal80*Δ	SL365	
SL1503	*gal80*Δ		[14]
SL1514	*IMA5-GFP*	SL229	
SL1530	*gal1-10-7*Δ	SL365	
SL1536	*gal1-10-7*Δ *gal80*Δ	SL1530	
SL1537	*gal80*Δ *ima1*Δ	SL1489	
SL1538	*ima1*Δ *IMA5-GFP*	SL1425	
SL1547	*ima1*Δ	SL365	
SL1556	*gal80*Δ *gal2*Δ	SL1489	
SL1558	*gal80*Δ *gal4*Δ	SL1489	
SL1566	*pCCW12-GAL2*	SL365	
SL1622	*pCCW12-MAL11*	SL365	
SL1624	*gal80*Δ *pCCW12-MAL11*	SL1489	
SL1626	*icl1*Δ		[14]

Table S1. Strains

Media	Component	Purpose	Reference
XY-glucose	10 g/L Bacto yeast extract; 20 g/L Bacto Peptone; 0.1 g/L adenine; 0.2 g/L tryptophan	To bring up strains from glycerol stock; outgrow after yeast transformation	
SC	1.4 g/L Edinburgh Amino Acids Mix (Formedium, EDI0100); 5 g/L $(NH_4)_2SO_4$; 1.7 g/L yeast nitrogen base without amino acids and ammonium; 50 mg/L adenine hemisulphate; 100 mg/L histidine; 100 mg/L methionine; 200 mg/L leucine; 100 mg/L tryptophan; 100 mg/L uracil	To set up pre-cultures; Run plate-reader experiments with auxotroph strains	
LoFlo	Same as SC, except that yeast nitrogen base without amino acids is LOFLO version (Formedium)	run plate reader experiments with prototroph strains	
Delft	3 g/L KH_2PO_4; 0.5 g/L $MgSO_4{\cdot}7H_2O$; 5 g/L $(NH_4)_2SO_4$; trace metals; vitamins	To run plate reader experiments with prototroph strains	[15, 16]
LB	10 g/L tryptone; 10 g/L NaCl; 5 g/L yeast extract	Grow *E. coli* for mini-prep	
SOC	2% (w/v) tryptone; 0.5% (w/v) yeast extract; 10 mM NaCl; 2.5 mM KCl; 10 mM $MgCl_2$; 20 mM glucose	For out-growth of *E. coli* after heat-shock transformation	

Table S2. Media

Plasmid ID	Plasmid name	Purpose	Reference
PLSM13	pYM40	gene deletion with *HPH* marker	[17]
PLSM82	pKT128	tagging a gene with GFP, and with *HIS* marker	[18]
PLSM72	pFA6a-natMX6	gene deletion with *natMX6* marker	[19]
	Amp1278	for amplifying the guide DNA that works with Amp1284	[20]
	Amp1284	providing a Cas9 cassette for CRISPR	[20]
	pYTK001	*Esp3I* assembly	[21]
	pYTK002	Part 1 for *BsaI* assembly	[21]
	pYTK010	Part 2 for *BsaI* assembly	[21]
	pYTK053	Part 4 for *BsaI* assembly	[21]
	pYTK072	Part 5 for *BsaI* assembly	[21]
	pYTK077	Part 6 for *BsaI* assembly	[21]
	pYTK088	Part 7 for *BsaI* assembly	[21]
	pYTK089	Part 8a for *BsaI* assembly	[21]
	pYTK094	Part 8b for *BsaI* assembly	[21]
HY03		*BsaI* assembly product to insert *pCCW12-GAL2* into the *HO* locus	
HY18		Gibson Assembly product to insert *pCCW12-MAL11* into the *HO* locus	

Table S3. Plasmids

Name	Version	Reference
FASTQC	0.11.9	
Cutadapt	1.18	[22]
HISAT2	2.1.0	[23]
SAMtools	1.11	[24]
BEDTools	2.30.0	[25]
Subread	2.0.0	[26]
MultiQC	1.13	[27]

Table S4. Versions of the software used in the RNA-seq pipeline

parameter	meaning	value	unit	reference
n	Hill number	2, 3, 4	dimensionless	
d_T	degradation rate of Mal11	0.01	min^{-1}	[10]
d_I	degradation rate of Ima1 and Ima5	0.027	min^{-1}	[7]
K_T	parameter in $u_T(p)$	1–10*	mM	
$u_{T,\max}$	maximum expression rate of *MAL11*	1	nM/min	[12]
b_T	basal expression rate of *MAL11*	0.01	nM/min	
K_I	parameter in $u_I(p)$	1	mM	[11]
$u_{I,\max}$	maximum expression rate of *IMA1/5*	*	nM/min	
b_I	basal expression rate of *IMA1/5*	0	nM/min	
$K_{m,I}$	Michaelis-Menten constant of Ima1/5	18	mM	[7]
$v_{I,\max}$	maximum reaction rate per unit of Ima1/5	4680	min^{-1}	[7]
v_T	import rate of palatinose per unit of Mal11	*	min^{-1}	

Table S5. Parameter values used in the mathematical model. The asterisk indicates the range used for bifurcation parameters.

variable or parameter	definition	parameter value
t'	$d_I t$	variable
p'	p/K_I	variable
I'	I/K_I	variable
T'	T/K_I	variable
d'_T	d_T/d_I	0.37
K'_T	K_T/K_I	bifurcation: 1–10
$u'_{T,\max}$	$u_{T,\max}/(K_I d_I)$	3.7×10^{-5}
b'_T	$b_T/(K_I d_I)$	3.7×10^{-7}
$u'_{I,\max}$	$u_{I,\max}/(K_I d_I)$	see α
b'_I	$b_I/(K_I d_I)$	0
$K'_{m,I}$	$K_{m,I}/K_I$	18
$v'_{I,\max}$	$v_{I,\max}/d_I$	1.7×10^5
v'_T	v_T/d_I	see α
α	$u'_{I,\max}/v'_T$	bifurcation: 0.6–5×10^{-9}

Table S6. Definition of the non-dimensionalised variables and parameters and the parameter values are calculated from the values in Table S5.

Supplementary Figures

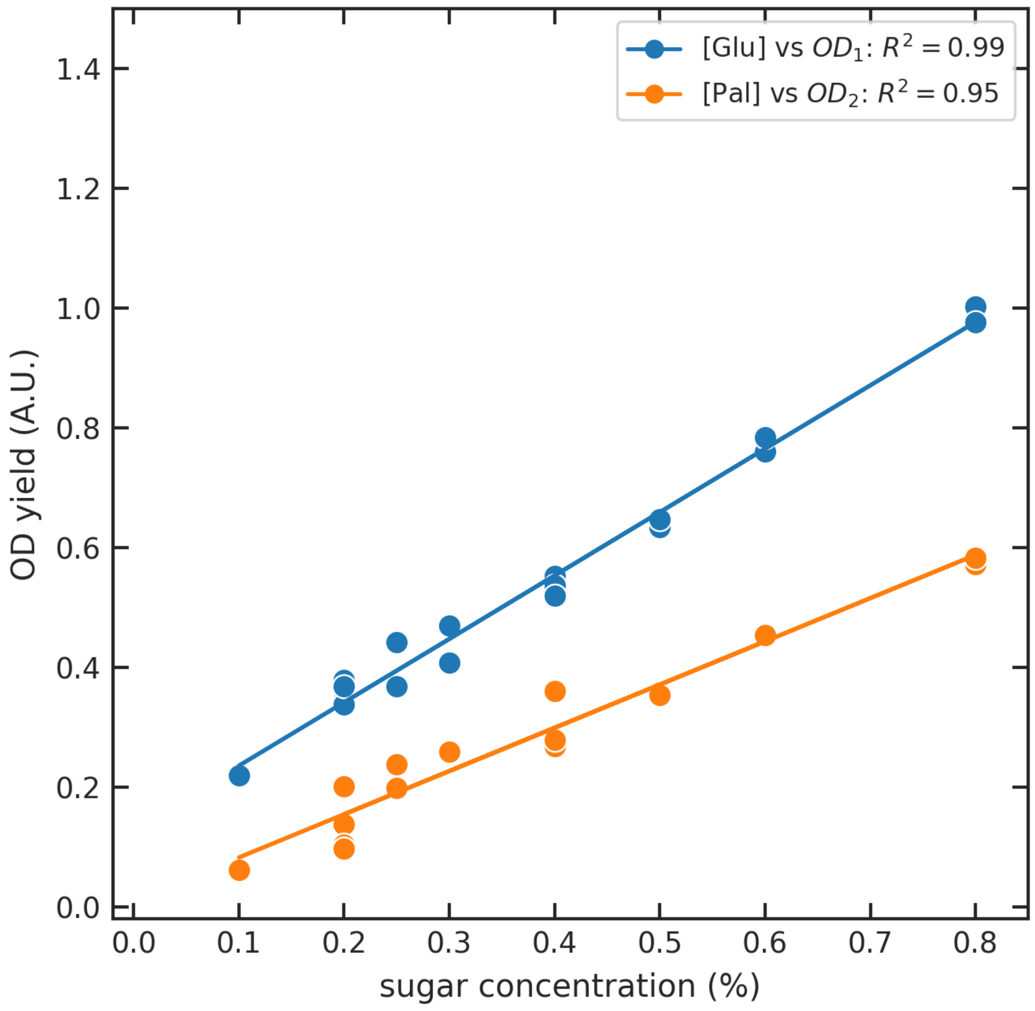

Figure S1. In glucose-palatinose mixtures, the OD yield of growth phase 1 (OD_1) linearly correlates with glucose concentrations, and the OD yield of growth phase 2 (OD_2) linearly correlates with palatinose concentrations. We found each data point using the method of Fig. 2C.

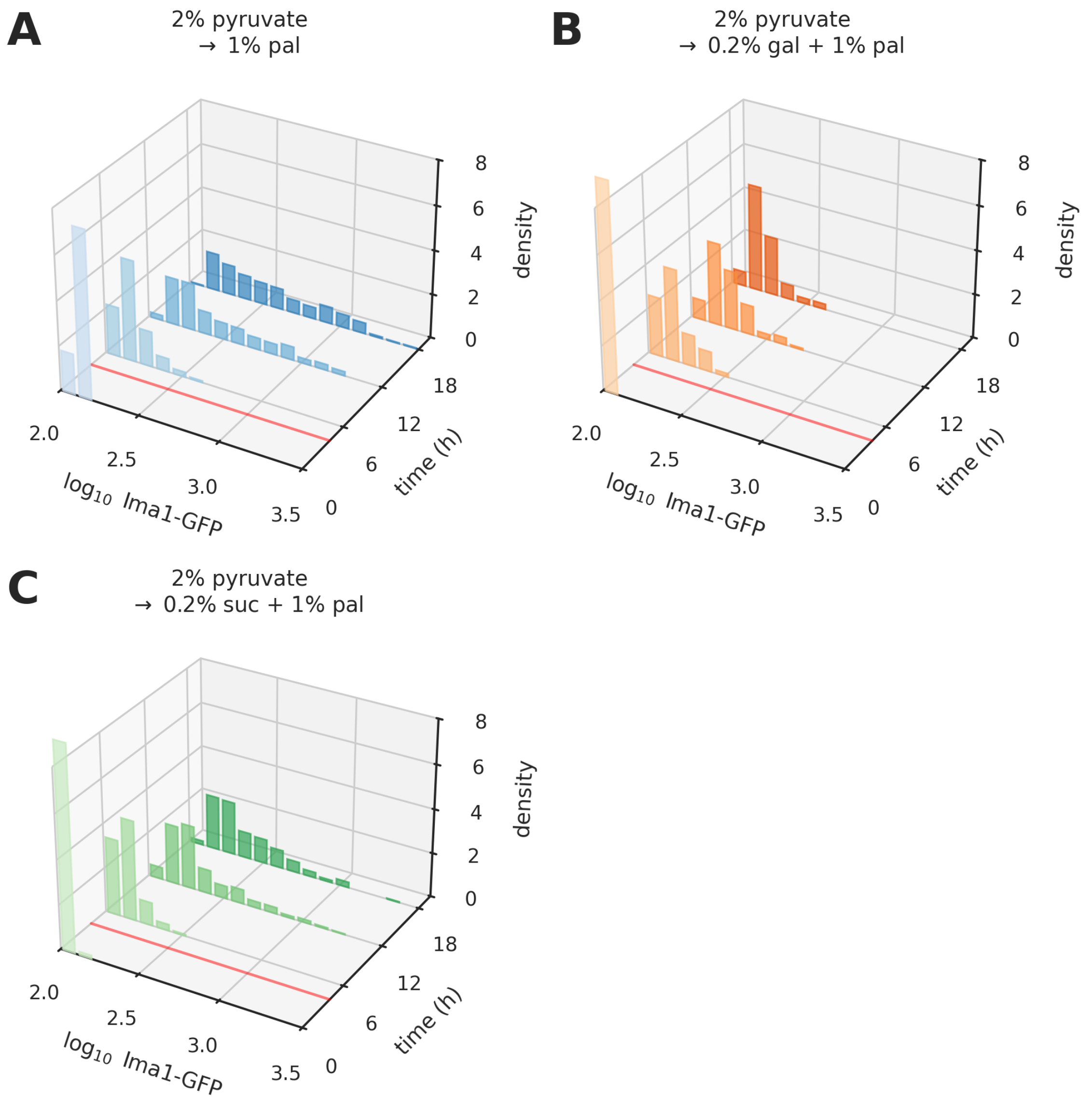

Figure S2. We used ALCATRAS microfluidic devices to monitor single-cell gene expression over time (see Supplementary Experimental Methods 1.3). The distribution of single-cell $\log_{10}$ Ima1-GFP signals at four time points (0, 6, 12 and 18 h). The red line shows when we switched the medium from 2% pyruvate to **(A)** 1% palatinose, **(B)** 0.2% galactose and 1% palatinose, and **(C)** 0.2% sucrose and 1% palatinose.

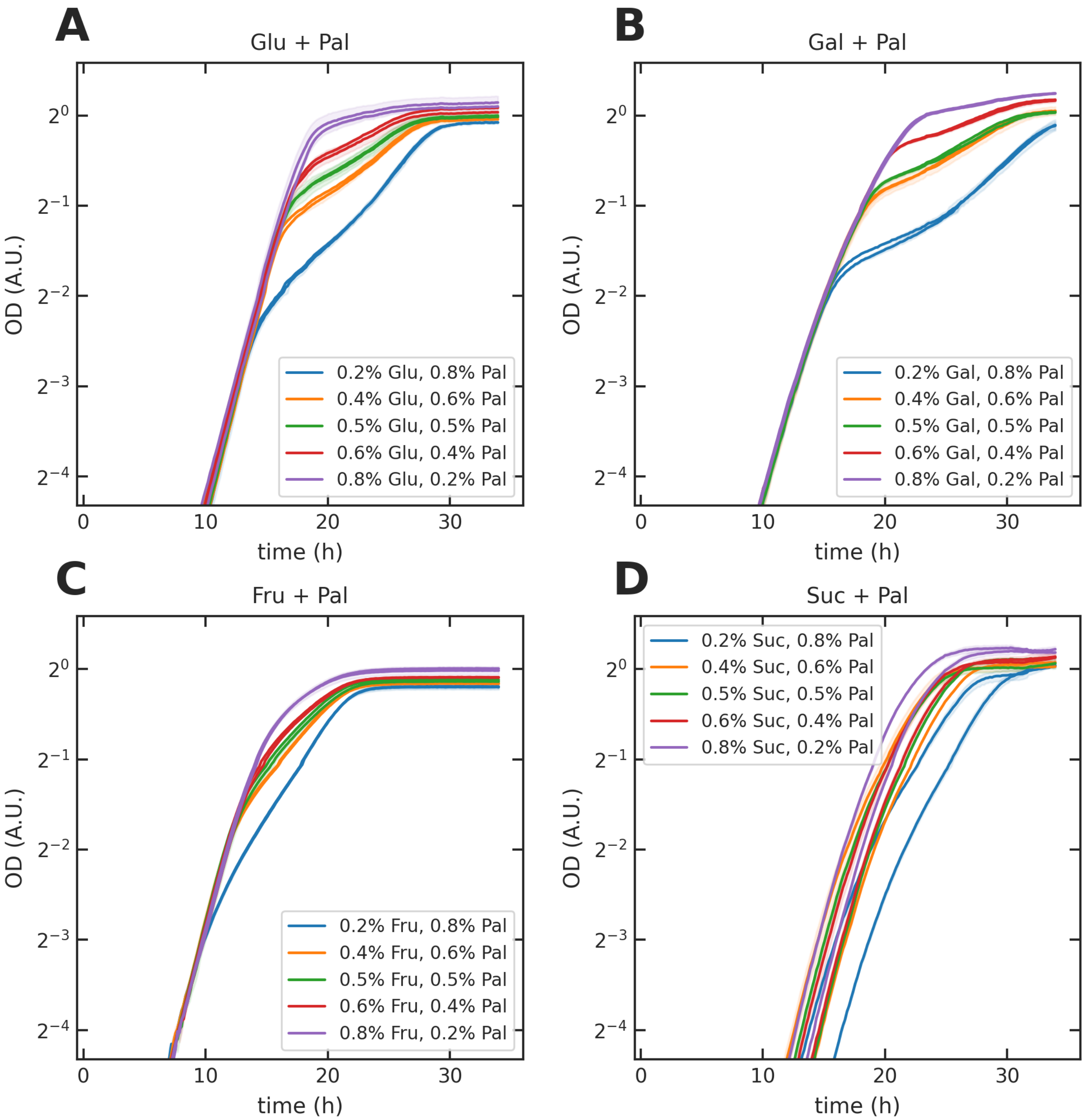

Figure S3. There is clear diauxie in glucose-palatinose and galactose-palatinose mixtures, but not with mixtures of sucrose or fructose. Each curve represents one biological replicate; the shading shows the standard deviation of two technical replicates. **(A–D)** The growth dynamics of the wild-type strain (FY4).

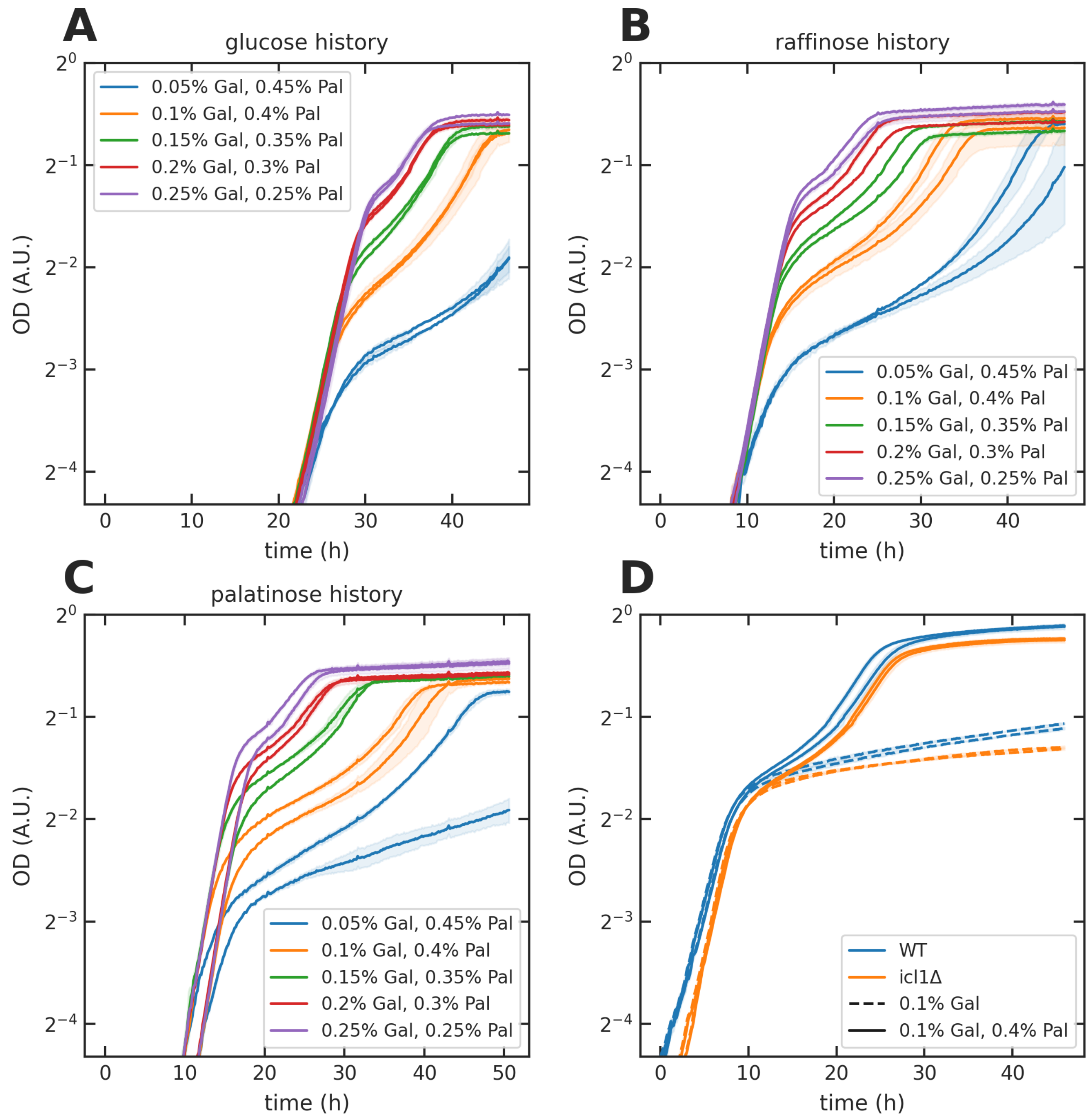

Figure S4. Diauxie in galactose-palatinose is independent of the pre-growth sugar. See Supplementary Experimental Methods 1.1 for the explanation of pre-growth history. Each curve represents one biological replicate; the shading shows the standard deviation of two technical replicates. **(A–C)** The growth dynamics of the wild-type (FY4) strain in galactose-palatinose mixtures with pre-growth in either glucose, raffinose, or palatinose. **(D)** Cells undergoing diauxie do not require extracellular acetate or ethanol. The growth dynamics of the wild-type strain (BY4741) and an *icl1*Δ mutant, which cannot metabolise C_2 substrates [28, 29].

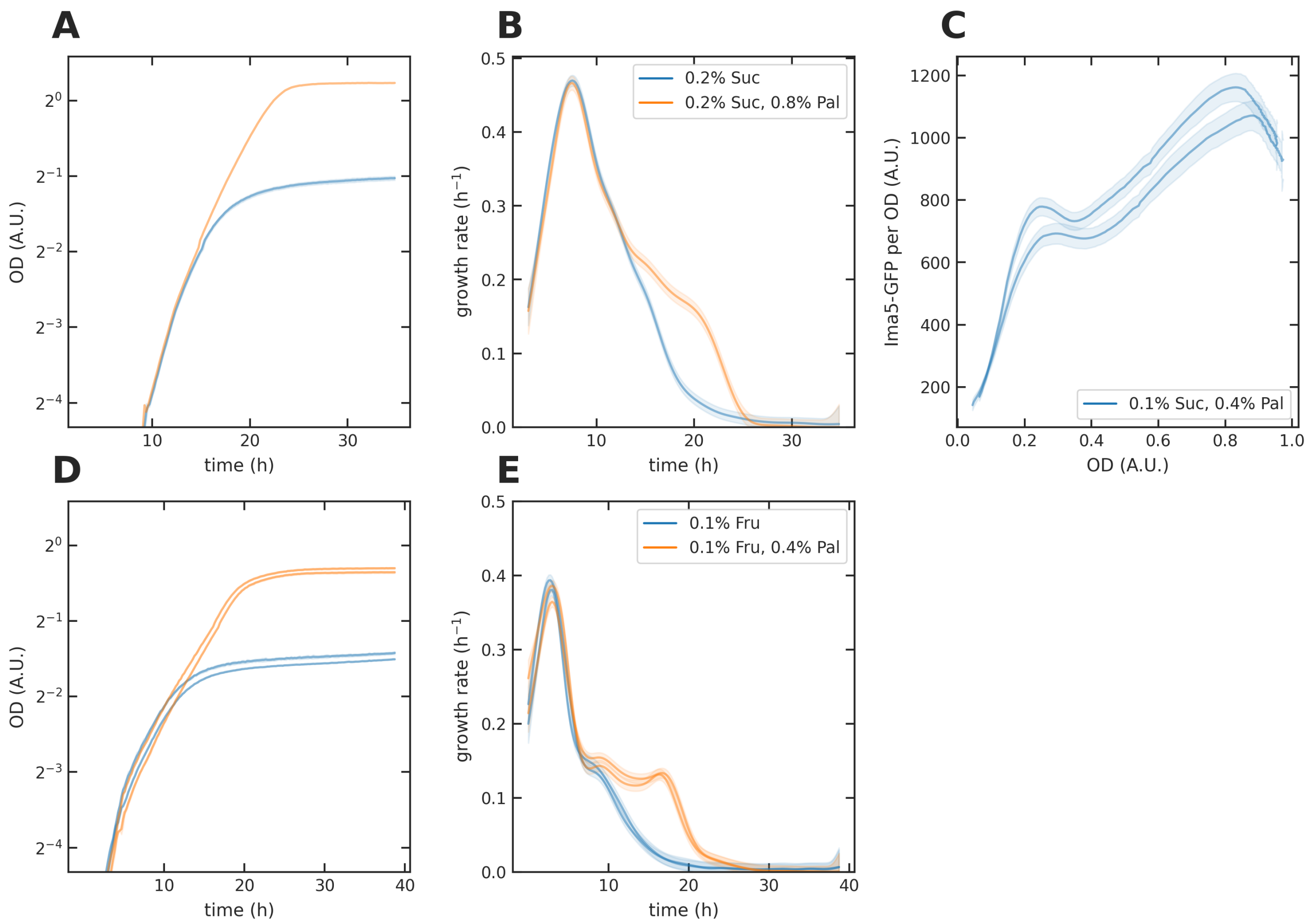

Figure S5. Growth on a sucrose-palatinose or fructose-palatinose mixture does not exhibit classic diauxie, but we observed a 'shoulder' in the growth rate over time. **(A)** The growth curve of an FY4 prototrophic strain in minimal medium with sucrose and palatinose is smooth with no obvious kinks. **(B)** Estimating the specific growth rate, however, shows a 'shoulder' for this choice of concentrations. There is no local minimum. **(C)** Cells derived from the BY4741 strain express Ima5-GFP immediately in the sucrose-palatinose mixture, not after a delay. **(D)** The growth curve of the BY4741 strain in LoFlo medium with fructose and palatinose is smooth with no obvious kinks. **(E)** Estimating the specific growth rate, however, shows a 'shoulder' for this choice of concentrations with no obvious local minimum. We show the fluorescence data from the same experiment in Fig. 3A.

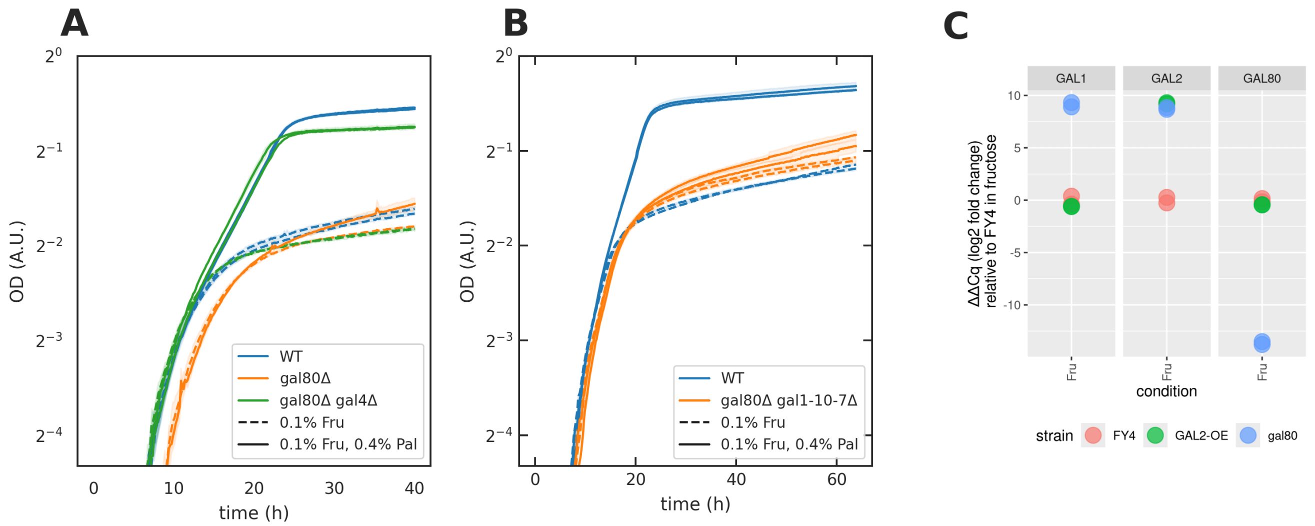

Figure S6. **(A, B)** The growth dynamics of the wild-type strain (FY4) and mutants in 0.1% fructose and 0.1% fructose + 0.4% palatinose. Each curve represents one biological replicate; the shading shows the standard deviation of two technical replicates. **(C)** qPCR results show that the transcript level of *GAL2* driven by the *CCW12* promoter is similar to that of *GAL2* in the *gal80*Δ mutant in 0.1% fructose. Each data point represents a biological replicate. The C_q value of each gene is the number of cycles required to amplify the input cDNA to a fixed threshold using a pair of primers targeting the gene. ΔC_q of each gene for each biological replicate is the difference between the C_q of the gene and the mean C_q of three reference genes (*ALG9*, *ACT1* and *PUS7*) from the same biological replicate. The $\Delta\Delta C_q$ of each gene is the difference between its ΔC_q and the mean ΔC_q of the gene in the FY4 wild-type strain. Cells were grown in 0.1% fructose and samples harvested at around OD 0.3. See Supplementary Experimental Methods 1.4 for detailed methods.

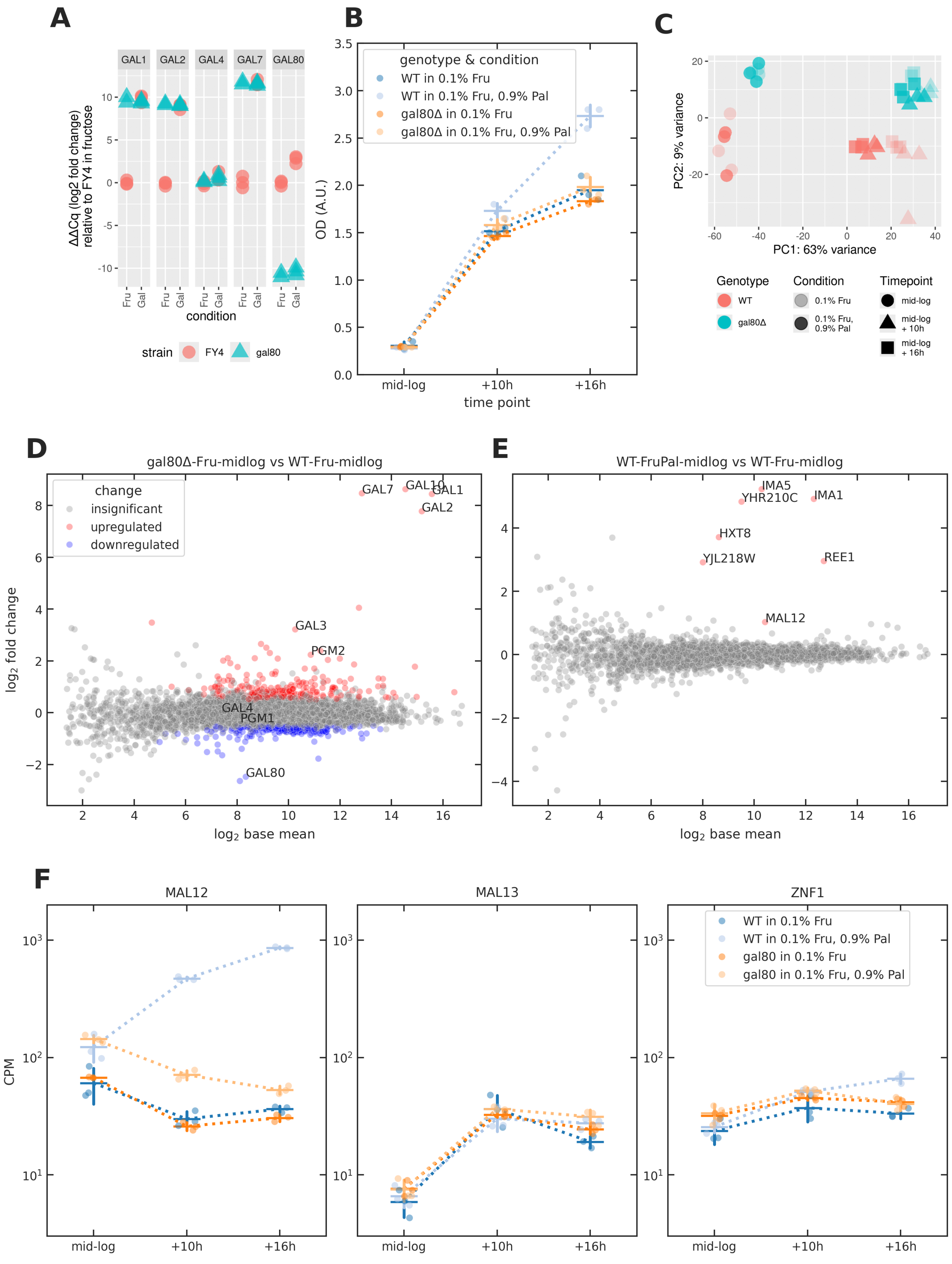
A
GAL1
GAL2
GAL4
GAL7
GAL80
ΔΔCq (log2 fold change) relative to FY4 in fructose
condition
Fru
Gal
strain
FY4
gal80
B
genotype & condition
WT in 0.1% Fru
WT in 0.1% Fru, 0.9% Pal
gal80Δ in 0.1% Fru
gal80Δ in 0.1% Fru, 0.9% Pal
OD (A.U.)
mid-log
+10h
+16h
time point
C
PC2: 9% variance
PC1: 63% variance
Genotype
WT
gal80Δ
Condition
0.1% Fru
0.1% Fru, 0.9% Pal
Timepoint
mid-log
mid-log + 10h
mid-log + 16h
D
gal80Δ-Fru-midlog vs WT-Fru-midlog
change
insignificant
upregulated
downregulated
GAL7
GAL10
GAL1
GAL2
GAL3
PGM2
GAL4
PGM1
GAL80
log2 fold change
log2 base mean
E
WT-FruPal-midlog vs WT-Fru-midlog
IMA5
YHR210C
IMA1
HXT8
YJL218W
REE1
MAL12
log2 base mean
F
MAL12
MAL13
ZNF1
CPM
WT in 0.1% Fru
WT in 0.1% Fru, 0.9% Pal
gal80 in 0.1% Fru
gal80 in 0.1% Fru, 0.9% Pal
mid-log
+10h
+16h

Figure S7 *(preceding page).* **(A)** qPCR results showing that the *GAL* transcript levels of the *gal80*Δ mutant in 0.1% fructose were similar to those in 0.1% galactose. The definition of a data point and $\Delta\Delta C_q$ is the same as Fig. S6C. See also Supplementary Experimental Methods 1.4 for detailed methods. **(B)** The growth curve of cells for RNA-seq showing the time points when sample were taken. Data are shown as mean ± standard deviation of three biological replicates (dots). **(C)** A principal component analysis (PCA) shows different expression between the wild-type and *gal80*Δ mutant and between the mid-log and later time points. We transformed the raw counts with a variance stabilising transformation (VST) before PCA and analysed counts of all 5697 genes. **(D)** A comparison between the transcriptome of the *gal80*Δ mutant and wild-type, both in 0.1% fructose and harvested at mid-log. **(E)** A comparison between the wild-type's transcriptome in 0.1% fructose and 0.9% palatinose at mid-log with the wild-type in 0.1% fructose also at mid-log. For (D) and (E), each panel plots the $\log_2$ fold change of each gene against its $\log_2$ base mean, both calculated with DESeq2 [30]. The base mean is the mean of the normalised counts of all samples in the pair of conditions that we are comparing; the counts are normalised by the sequencing depth. We label the genes that are significantly up- and down-regulated red and blue. The criteria for significance are $|\log_2 \text{fold change}| > 0.5$ with the adjusted p-value smaller than 0.05. **(F)** The count per million reads (CPM) of *MAL12*, *MAL13* and *ZNF1* transcripts. The error bar shows the standard deviation of three technical replicates. Data are shown as mean ± standard deviation of three biological replicates (dots).

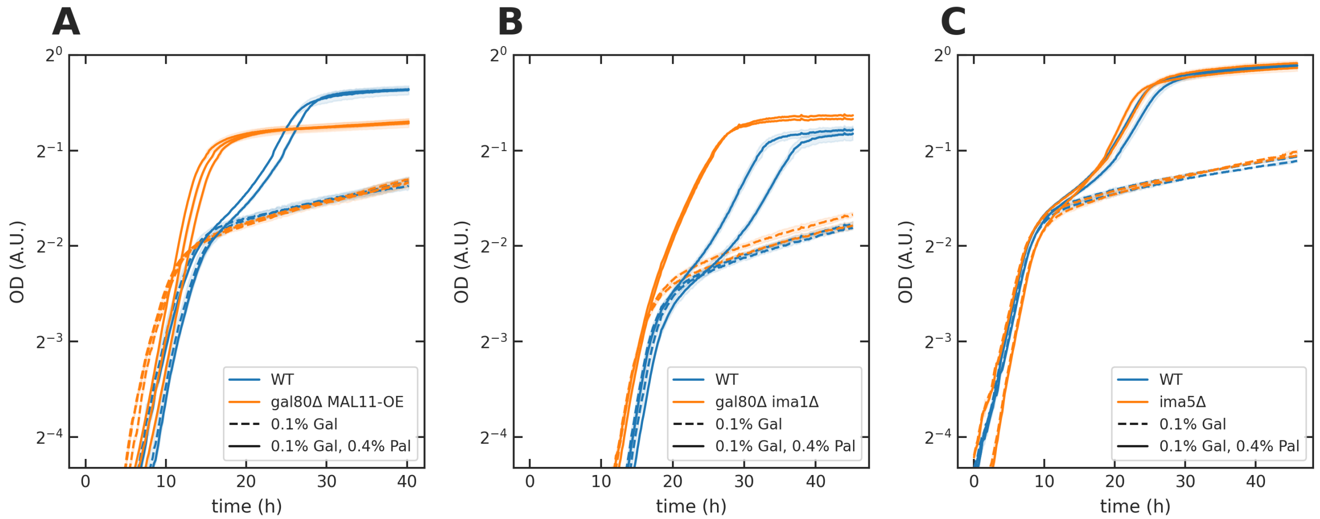

Figure S8. **(A)** The growth dynamics of the wild-type strain (FY4) and the *gal80*Δ *MAL11* over-expression (OE) strain driven by the CCW12 promoter. **(B)** The growth dynamics of the wild-type strain (FY4) and the *gal80*Δ *ima1*Δ mutant. **(C)** The growth dynamics of the wild-type strain (BY4741) and the *ima5*Δ mutant. We use dashed lines to indicate single sugars, full lines to indicate mixtures, and each curve represents one biological replicate; the shading shows the standard deviation of two technical replicates.

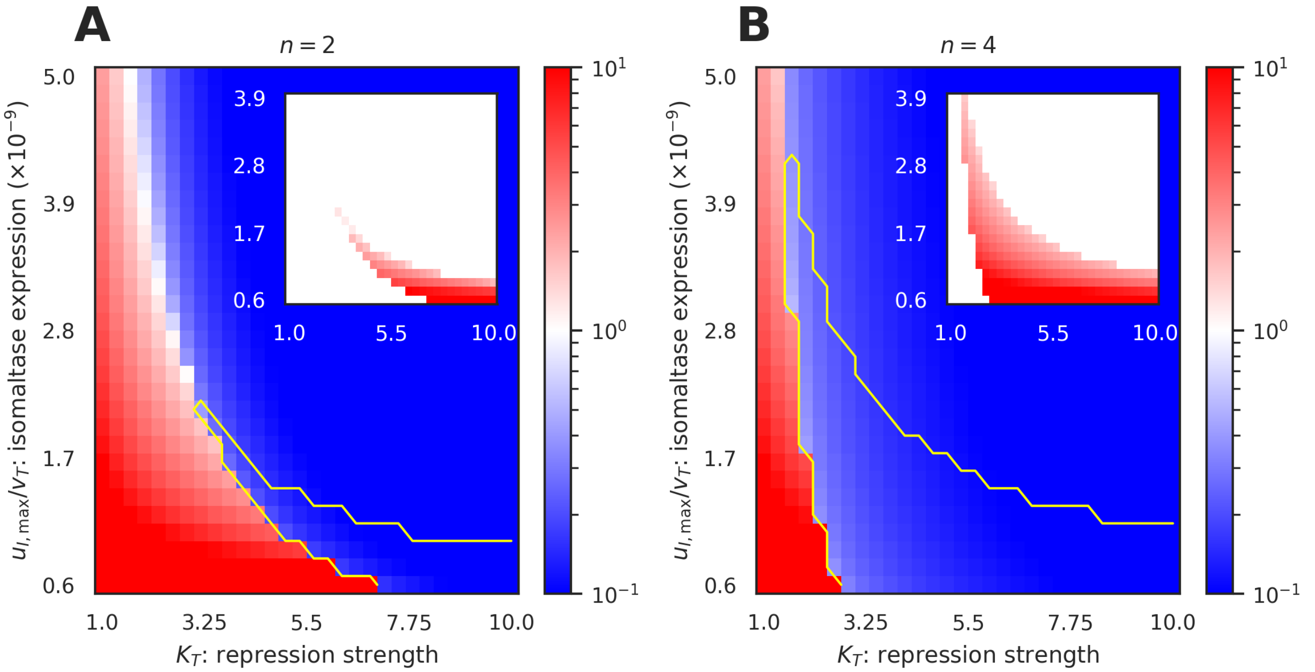

Figure S9. The steady-state p/K_T value as a function of the repression strength, K_T, and the ratio of maximal *IMA* expression to the palatinose import rate, $u_{I,\max}/v_T$ for Hill numbers **(A)** $n = 2$ and **(B)** $n = 4$. We define the system to be ON if $p/K_T > 1$ (red). The yellow contour marks a bistable region; the inset shows the value of p/K_T at the high steady state. Parameter values are in Table S6. The ratio $u_{I,\max}/v_T$ has a minimal value to prevent any steady states with infinite p, which we presume evolution avoids.

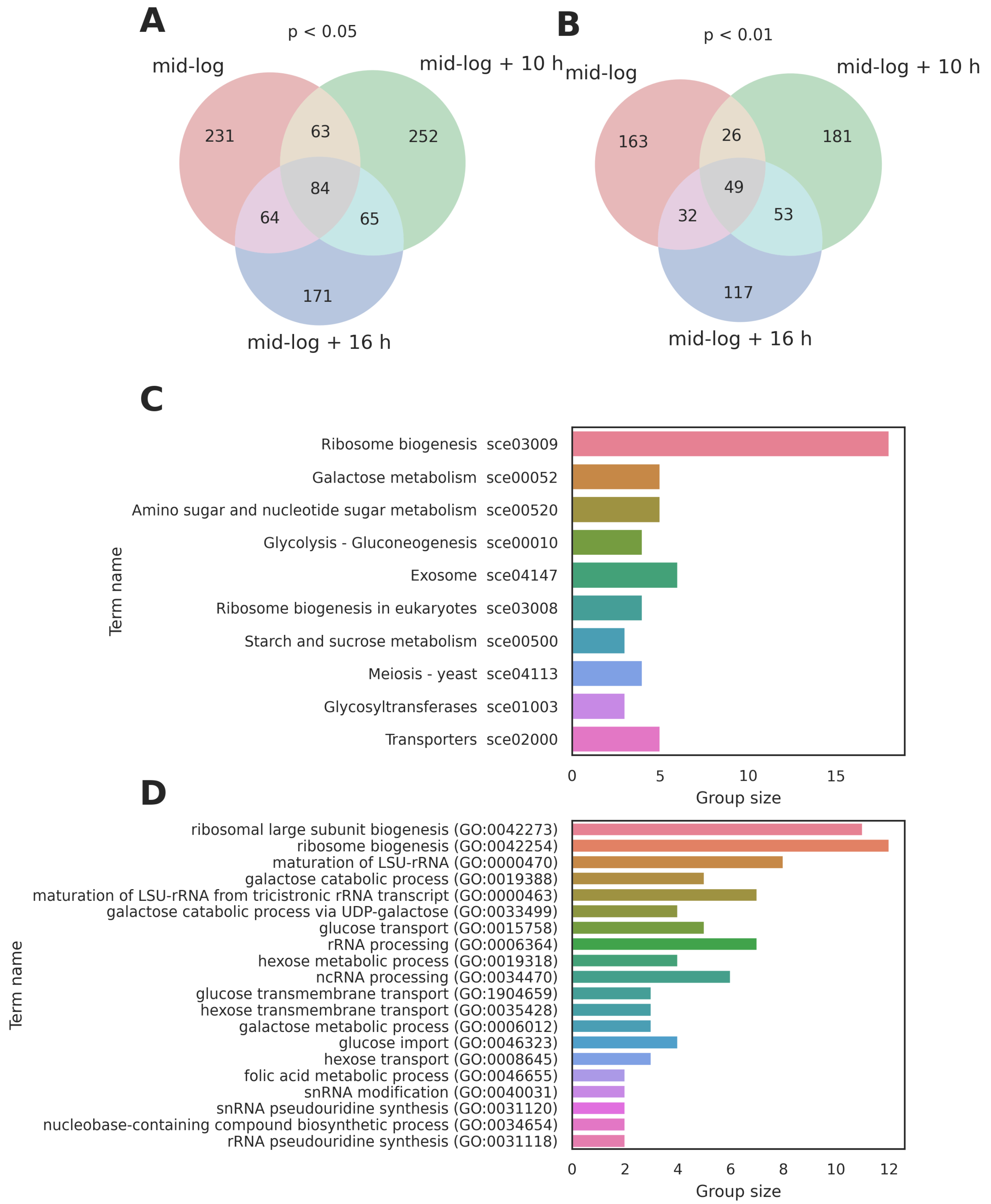
A
p < 0.05
mid-log
mid-log + 10 h
mid-log + 16 h
231
63
252
84
64
65
171
B
p < 0.01
mid-log
mid-log + 10 h
mid-log + 16 h
163
26
181
49
32
53
117
C
Term name
Ribosome biogenesis sce03009
Galactose metabolism sce00052
Amino sugar and nucleotide sugar metabolism sce00520
Glycolysis - Gluconeogenesis sce00010
Exosome sce04147
Ribosome biogenesis in eukaryotes sce03008
Starch and sucrose metabolism sce00500
Meiosis - yeast sce04113
Glycosyltransferases sce01003
Transporters sce02000
0
5
10
15
Group size
D
Term name
ribosomal large subunit biogenesis (GO:0042273)
ribosome biogenesis (GO:0042254)
maturation of LSU-rRNA (GO:0000470)
galactose catabolic process (GO:0019388)
maturation of LSU-rRNA from tricistronic rRNA transcript (GO:0000463)
galactose catabolic process via UDP-galactose (GO:0033499)
glucose transport (GO:0015758)
rRNA processing (GO:0006364)
hexose metabolic process (GO:0019318)
ncRNA processing (GO:0034470)
glucose transmembrane transport (GO:1904659)
hexose transmembrane transport (GO:0035428)
galactose metabolic process (GO:0006012)
glucose import (GO:0046323)
hexose transport (GO:0008645)
folic acid metabolic process (GO:0046655)
snRNA modification (GO:0040031)
snRNA pseudouridine synthesis (GO:0031120)
nucleobase-containing compound biosynthetic process (GO:0034654)
rRNA pseudouridine synthesis (GO:0031118)
0
2
4
6
8
10
12
Group size

Figure S10 ***(preceding page).*** We defined the set of differentially expressed genes (DEGs) between the wild-type and the *gal80*Δ mutant in 0.1% fructose by those statistically significant, present over all three time points, and with a $|\log_2 \text{fold change}|$ larger than 0.5. A Venn diagram showing the number of these genes when the threshold for significance for the adjusted p-value is **(A)** 0.05 and **(B)** 0.01. Both the adjusted p-value and the $\log_2$ fold change are calculated with DESeq2 [30]. **(C)** The enrichment on gene ontology (GO) biological process for the genes in (A). The group size of each term is the number of genes that are in both our set of DEGs and the GO term. We used YeastEnrichR to perform the enrichment [31]. Only GO terms whose group size ≥ 2 and adjusted p-value ≤ 0.01 are shown, with the terms ranked by their adjusted p-value and only the top 10 displayed. **(D)** Pathway enrichment for the genes in (A) using the Kyoto Encyclopedia of Genes and Genomes (KEGG) database. The group size of each term is the number of genes that are in both our set of DEGs and the GO term. We used YeastEnrichR to perform the enrichment [31]. Only pathway terms whose group size ≥ 2 and adjusted p-value < 0.01 are shown, ranked by their adjusted p-value.

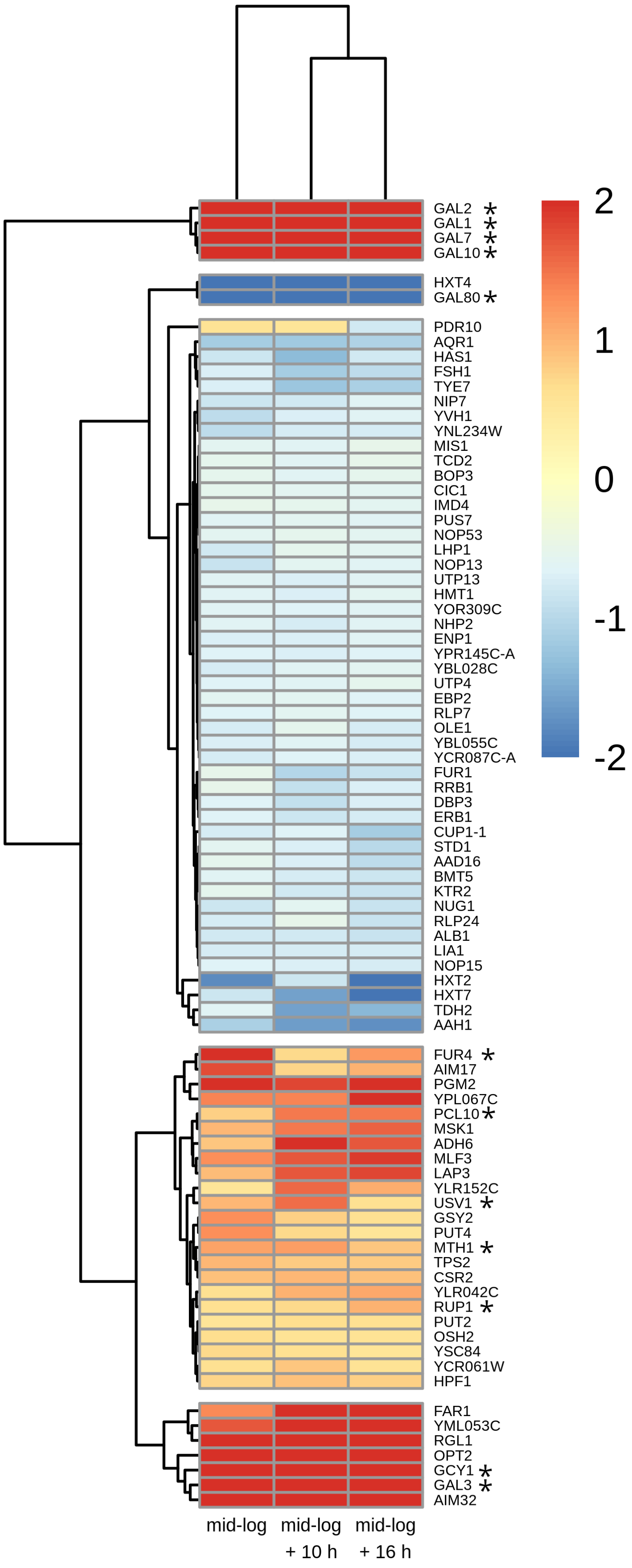

GAL2 *
GAL1 *
GAL7 *
GAL10 *
HXT4
GAL80 *
PDR10
AQR1
HAS1
FSH1
TYE7
NIP7
YVH1
YNL234W
MIS1
TCD2
BOP3
CIC1
IMD4
PUS7
NOP53
LHP1
NOP13
UTP13
HMT1
YOR309C
NHP2
ENP1
YPR145C-A
YBL028C
UTP4
EBP2
RLP7
OLE1
YBL055C
YCR087C-A
FUR1
RRB1
DBP3
ERB1
CUP1-1
STD1
AAD16
BMT5
KTR2
NUG1
RLP24
ALB1
LIA1
NOP15
HXT2
HXT7
TDH2
AAH1
FUR4 *
AIM17
PGM2
YPL067C
PCL10 *
MSK1
ADH6
MLF3
LAP3
YLR152C
USV1 *
GSY2
PUT4
MTH1 *
TPS2
CSR2
YLR042C
RUP1 *
PUT2
OSH2
YSC84
YCR061W
HPF1
FAR1
YML053C
RGL1
OPT2
GCY1 *
GAL3 *
AIM32
mid-log
mid-log + 10 h
mid-log + 16 h
2
1
0
-1
-2

Figure S11 ***(preceding page).*** 83 out of the 84 differentially expressed genes identified in Fig. S10A were consistently up- or down-regulated over all three time points. The colour shows the $\log_2$ fold change of each gene at each time point. We used complete-linkage clustering and the Euclidean distance between rows or columns to perform the hierarchical clustering. The asterisk (*) indicates that the gene's promoter is known to be directly bound by Gal4 [32, 33].

References

[1] Gietz, R. D. and Woods, R. A. Transformation of yeast by lithium acetate/single-stranded carrier DNA/polyethylene glycol method. *Meth. Enzymol.* **350**, 87–96 (2002).

[2] Crane, M. M., Clark, I. B., Bakker, E., Smith, S., and Swain, P. S. A microfluidic system for studying ageing and dynamic single-cell responses in budding yeast. *PLoS ONE* **9**(6), 1–10 (2014).

[3] Pietsch, J. M., Muñoz, A. F., Adjavon, D.-Y. A., Farquhar, I., Clark, I. B., and Swain, P. S. Determining growth rates from bright-field images of budding cells through identifying overlaps. *eLife* **12**, e79812, July (2023).

[4] Barrass, J. D., Reid, J. E. A., Huang, Y., Hector, R. D., Sanguinetti, G., Beggs, J. D., and Granneman, S. Transcriptome-wide RNA processing kinetics revealed using extremely short 4tU labeling. *Genome Biol.* **16**(1), 282, December (2015).

[5] Wallace, E. W. J. and Haynes, S. J. Tidyqpcr: Quantitative PCR analysis in the tidyverse. *J. Open Source Softw.* **7**(74), 4507, June (2022).

[6] Brown, C. A., Murray, A. W., and Verstrepen, K. J. Rapid Expansion and Functional Divergence of Subtelomeric Gene Families in Yeasts. *Curr. Biol.* **20**(10), 895–903 (2010).

[7] Deng, X., Petitjean, M., Teste, M.-A., Kooli, W., Tranier, S., François, J. M., and Parrou, J.-L. Similarities and differences in the biochemical and enzymological properties of the four isomaltases from Saccharomyces cerevisiae. *FEBS Open Bio* **4**(1), 200–212 (2014).

[8] Kim, J.-R., Yoon, Y., and Cho, K.-H. Coupled Feedback Loops Form Dynamic Motifs of Cellular Networks. *Biophys. J.* **94**(2), 359–365, January (2008).

[9] Tian, X.-J., Zhang, X.-P., Liu, F., and Wang, W. Interlinking positive and negative feedback loops creates a tunable motif in gene regulatory networks. *Phys. Rev. E* **80**(1), 011926, July (2009).

[10] Alonso, A. and Kotyk, A. Apparent half-lives of sugar transport proteins in Saccharomyces cerevisiae. *Folia Microbiol.* **23**(2), 118–125, March (1978).

[11] Hatanaka, H. . PhD thesis, Osaka University, (2018).

[12] Venturelli, O. S., El-Samad, H., and Murray, R. M. Synergistic dual positive feedback loops established by molecular sequestration generate robust bimodal response. *Proc. Natl. Acad. Sci. U.S.A.* **109**(48), E3324–E3333 (2012).

[13] Baker Brachmann, C., Davies, A., Cost, G. J., Caputo, E., Li, J., Hieter, P., and Boeke, J. D. Designer deletion strains derived from Saccharomyces cerevisiae S288C: A useful set of strains and plasmids for PCR-mediated gene disruption and other applications. *Yeast* **14**(2), 115–132 (1998).

[14] Winzeler, E. A., Shoemaker, D. D., Astromoff, A., Liang, H., Anderson, K., Andre, B., Bangham, R., Benito, R., Boeke, J. D., Bussey, H., Chu, A. M., Connelly, C., Davis, K., Dietrich, F., Dow, S. W., El Bakkoury, M., Foury, F., Friend, S. H., Gentalen, E., Giaever, G., Hegemann, J. H., Jones, T., Laub, M., Liao, H., Liebundguth, N., Lockhart, D. J., Lucau-Danila, A., Lussier, M., M'Rabet, N., Menard, P., Mittmann, M., Pai, C., Rebischung, C., Revuelta, J. L., Riles, L., Roberts, C. J., Ross-MacDonald, P., Scherens, B., Snyder, M., Sookhai-Mahadeo, S., Storms, R. K., Véronneau, S., Voet, M., Volckaert, G., Ward, T. R., Wysocki, R., Yen, G. S., Yu, K., Zimmermann, K., Philippsen, P., Johnston, M., and Davis, R. W. Functional Characterization of the S. cerevisiae Genome by Gene Deletion and Parallel Analysis. *Science* **285**(5429), 901–906, August (1999).

[15] Verduyn, C., Postma, E., Scheffers, W. A., and van Dijken, J. P. . Physiology of Saccharomyces cerevisiae in anaerobic glucose-limited chemostat cultures. *Microbiology* **136**(3), 395–403 (1990).

[16] Verduyn, C., Postma, E., Scheffers, W. A., and Van Dijken, J. P. Effect of benzoic acid on metabolic fluxes in yeasts: A continuous-culture study on the regulation of respiration and alcoholic fermentation. *Yeast* **8**(7), 501–517 (1992).

[17] Janke, C., Magiera, M. M., Rathfelder, N., Taxis, C., Reber, S., Maekawa, H., Moreno-Borchart, A., Doenges, G., Schwob, E., Schiebel, E., and Knop, M. A versatile toolbox for PCR-based tagging of yeast genes: New fluorescent proteins, more markers and promoter substitution cassettes. *Yeast* **21**(11), 947 (2004).

[18] Sheff, M. A. and Thorn, K. S. Optimized cassettes for fluorescent protein tagging in Saccharomyces cerevisiae. *Yeast* **21**(8), 661–670 (2004).

[19] Hentges, P., Van Driessche, B., Tafforeau, L., Vandenhaute, J., and Carr, A. M. Three novel antibiotic marker cassettes for gene disruption and marker switching in Schizosaccharomyces pombe. *Yeast* **22**(13), 1013–1019, October (2005).

[20] Shaw, W. M., Yamauchi, H., Mead, J., Gowers, G.-O. F., Bell, D. J., Öling, D., Larsson, N., Wigglesworth, M., Ladds, G., and Ellis, T. Engineering a Model Cell for Rational Tuning of GPCR Signaling. *Cell* **177**(3), 782–796.e27, April (2019).

[21] Lee, M. E., DeLoache, W. C., Cervantes, B., and Dueber, J. E. A Highly Characterized Yeast Toolkit for Modular, Multipart Assembly. *ACS Synth. Biol.* **4**(9), 975–986, September (2015).

[22] Martin, M. Cutadapt removes adapter sequences from high-throughput sequencing reads. *EMBnet.journal* **17**(1), 10–12, May (2011).

[23] Kim, D., Paggi, J. M., Park, C., Bennett, C., and Salzberg, S. L. Graph-based genome alignment and genotyping with HISAT2 and HISAT-genotype. *Nat. Biotechnol.* **37**(8), 907–915, August (2019).

[24] Li, H., Handsaker, B., Wysoker, A., Fennell, T., Ruan, J., Homer, N., Marth, G., Abecasis, G., Durbin, R., and 1000 Genome Project Data Processing Subgroup. The Sequence Alignment/Map format and SAMtools. *Bioinformatics* **25**(16), 2078–2079, August (2009).

[25] Quinlan, A. R. and Hall, I. M. BEDTools: A flexible suite of utilities for comparing genomic features. *Bioinformatics* **26**(6), 841–842, March (2010).

[26] Liao, Y., Smyth, G. K., and Shi, W. featureCounts: An efficient general purpose program for assigning sequence reads to genomic features. *Bioinformatics* **30**(7), 923–930, April (2014).

[27] Ewels, P., Magnusson, M., Lundin, S., and Käller, M. MultiQC: Summarize analysis results for multiple tools and samples in a single report. *Bioinformatics* **32**(19), 3047–3048, October (2016).

[28] Fernández, E., Moreno, F., and Rodicio, R. The ICL1 gene from Saccharomyces cerevisiae. *Eur. J. Biochem.* **204**(3), 983–990 (1992).

[29] Lee, Y. J., Jang, J. W., Kim, K. J., and Maeng, P. J. TCA cycle-independent acetate metabolism via the glyoxylate cycle in Saccharomyces cerevisiae. *Yeast* **28**(2), 153–166 (2011).

[30] Love, M. I., Huber, W., and Anders, S. Moderated estimation of fold change and dispersion for RNA-seq data with DESeq2. *Genome Biol.* **15**(12), 550, December (2014).

[31] Kuleshov, M. V., Jones, M. R., Rouillard, A. D., Fernandez, N. F., Duan, Q., Wang, Z., Koplev, S., Jenkins, S. L., Jagodnik, K. M., Lachmann, A., McDermott, M. G., Monteiro, C. D., Gundersen, G. W., and Ma'ayan, A. Enrichr: A comprehensive gene set enrichment analysis web server 2016 update. *Nucleic Acids Research* **44**(W1), W90–97, July (2016).

[32] Ren, B., Robert, F., Wyrick, J. J., Aparicio, O., Jennings, E. G., Simon, I., Zeitlinger, J., Schreiber, J., Hannett, N., Kanin, E., Volkert, T. L., Wilson, C. J., Bell, S. P., and Young, R. A. Genome-Wide Location and Function of DNA Binding Proteins. *Science* **290**(5500), 2306–2309, December (2000).

[33] Rhee, H. S. and Pugh, B. F. Comprehensive Genome-wide Protein-DNA Interactions Detected at Single-Nucleotide Resolution. *Cell* **147**(6), 1408–1419, December (2011).
