# Supplementary material for: The type of carbon source not the growth rate it supports can determine diauxie in Saccharomyces cerevisiae
Source: Commun Biol. 2025 Feb 27;8:325. doi: 10.1038/s42003-025-07747-z (PMC11868555; doi:10.1038/s42003-025-07747-z)
Supplement: Supplementary file 2 — Description of Additional Supplementary Files [file 42003_2025_7747_MOESM2_ESM.pdf]

Description of Additional Supplementary Data: The type of carbon source not the growth rate it supports can determine diauxie in *Saccharomyces cerevisiae*

Y Huo, W Danecka, I Farquhar, K Mailliet, T Moses, EWJ Wallace, PS Swain.

Supplementary Data 1

Filename: `Supplementary_Data_1.xlsx`

Description: All oligos used in this study for strain construction and qPCR.
